# Molecular Tools for Precision Targeting and Detection of G-Quadruplex Structures

**DOI:** 10.3390/molecules30204099

**Published:** 2025-10-15

**Authors:** Daniele Esposito, Alessandra Locatelli, Rita Morigi

**Affiliations:** Department of Pharmacy and Biotechnology, Alma Mater Studiorum—University of Bologna, Via Belmeloro 6, 40126 Bologna, Italy; daniele.esposito6@unibo.it (D.E.); alessandra.locatelli@unibo.it (A.L.)

**Keywords:** G-quadruplex, molecular tools, fluorescent probes, metal complexes, conjugates

## Abstract

In the context of the study of G-quadruplex (G4), the main field of focus is usually referred to binding molecules able to interact with these non-canonical conformations and stabilizing them leading to diverse biological effects. Although the cellular events triggered by these ligands are useful for potential anticancer applications, the development of innovative molecular tools to gain new information about G4 has become more urgent. The concept of G4-interacting molecular tools refers to chemical entities that can bind and interact with G-rich sequences of the genome—like traditional ligands—but simultaneously provide external outputs that can be interpreted and studied to obtain insights on their dynamics, position in the cellular context and more. Starting from traditional chemical approaches, researchers have worked to produce sophisticated and complex synthetic strategies in order to introduce more accurate instruments for their aims. This review provides a comprehensive and up-to-date overview of this research area by detailing the major classes of molecular tools, describing the latest updates about three main classes: small-molecules fluorescence probes, G4-binding metal complexes, and products of conjugation strategies. Overall, advancements in molecular tools targeting G4s have made the study of G4 formation, dynamics, and functions much easier; thus, increasing the knowledge of G4 biology, and creating new opportunities for biomedical and therapeutic applications, ultimately highlighting the importance of the development of molecular tools in G4 research.

## 1. Introduction

The study of genomic DNA features is crucial to better understand the biological events that happen at a cellular level. Among the main aspects, gaining information on the structural arrangement of this macromolecule and how its single components interact with each other can be key to gaining deeper insight on the role of nucleic acids in both physiological and pathological pathways. The high molecular weight and the structural complexity of DNA [1] have challenged the efforts of gaining knowledge on the overall topology inside the nucleus and determining how DNA secondary structures can change through the different biological processes. It is known that genomic DNA is a macromolecule whose most common conformation can be described by the double-stranded helix model, corresponding to the so-called B-DNA, i.e., a high stability and energy-favored condition in which it can be mainly found [2]. However, the dynamic environment that surrounds the nucleic acid and that builds up the genomic machinery is responsible for variations in the coiling and its conformation, making possible the observation of different secondary structures [3]. It has been shown that a higher number of non-canonical conformations can be seen during replication phases of the cell cycle, and negative supercoiling due to the unwinding of the double helix, together with the crowded molecular environment, can increase the chance of non-canonical topologies [4,5,6]. The result of all these variables is that about 13% of the human genome can be found in non-B-DNA forms [7] that have been screened and studied through the years, with insights obtained through characterization techniques such as NMR spectroscopy [8], circular dichroism (CD) [9], X-ray crystallography [10], and molecular dynamics [11]. Among the most known non-canonical structures, Z-DNA, cruciform DNA, i-motifs, and hairpins can be named, together with G-quadruplex structures [12,13,14].

**Figure 1:**
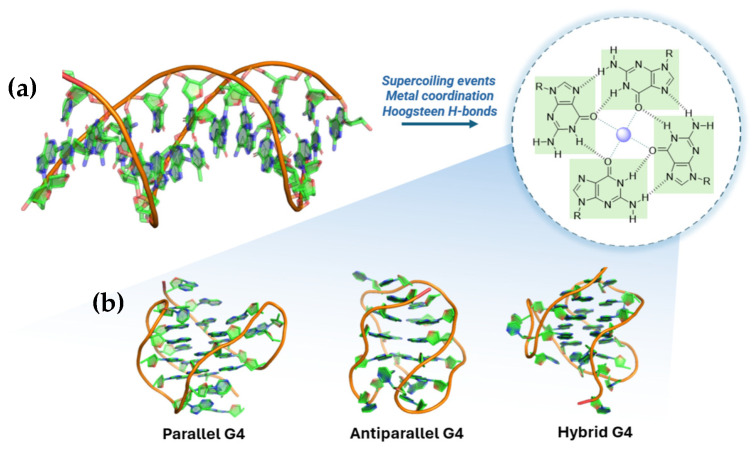
Schematic representation of G-quadruplex formation and topology. (**a**) Three-dimensional arrangement of canonical duplex DNA and G-quartet formation favored by supercoiling, metal coordination, and Hoogsteen hydrogen bonds. (**b**) Three common G-quadruplex topologies are represented: parallel, antiparallel, and hybrid. (PDB: 2HY9, 143D, 2JPZ).

More specifically, the term G-quadruplex (G4) stands for a guanine-enriched secondary structure in both DNA and RNA [15], characterized by a four-stranded filament obtained by the stacking of two or more guanine quartets. These guanine bases form a square co-planar configuration, thanks to Hoogsteen hydrogen bonds between the oxygen O6 of the carbonyl group and the amine hydrogen N1, and between nitrogen N7 and amine hydrogen N2. The formation of the structure also strictly depends on the coordination of carbonyl oxygen O6 with a metal cation, commonly monovalent cations like Na^+^, which is small enough to fit in the inner channel of the G4, and K^+^, which is larger and is located in the “interplane cavity” between G-tetrads [11]. Typically, G4s can be found in various conformations that vary greatly from each other, due to their polymorphism. For instance, intramolecular quadruplexes are included in the same single-stranded DNA, while intermolecular quadruplex refers to structures formed between two (bimolecular) or four (tetramolecular) strands. Either polymorph can present different topologies, such as parallel, antiparallel, and hybrid structures, depending on the length and composition of the loops, number of quartets, and the identity of the cations (Figure 1) [14,15,16].

G4 structures are present and conserved in various regions of the human genome such as chromosome ends (i.e., telomeres), gene promoter sequences, gene transcriptional regulatory regions, and DNA replication origins [17,18]. This correlates to the pivotal function of G4s in different biological processes as well as in pathological pathways, i.e., neurological disease and neoplastic events [19,20]. More than 700,000 G4 motifs were recognized in human cancerous cells [21,22], highlighting the presence of G4-forming sequences at the Transcription Start Sites (TSSs) of genes related to cancer, such as *c-MYC*, *BCL-2*, *KRAS*, *c-KIT*, and *VEGF* [12,23,24,25,26], and apart from the assessed role of G4 inside human cells, their relevance can also be linked to their presence in other biological environments, including viruses [27], bacteria [28], and other parasites [29], making them a useful and versatile target to gain insight into its pharmacological effects towards infections.

**Figure 2:**
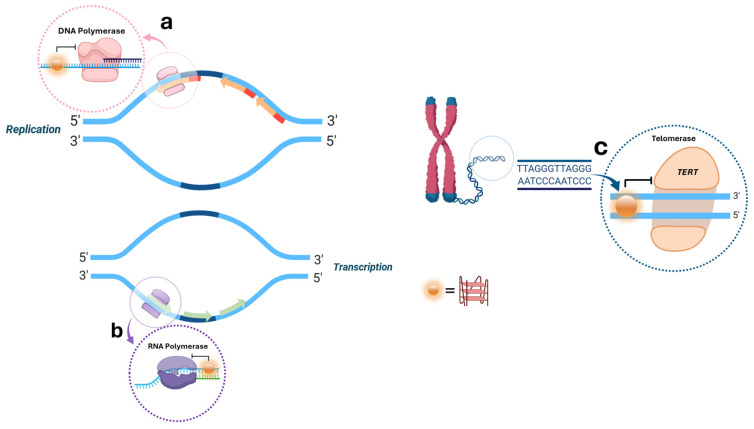
Biological roles of G4 structures during the main cellular events of replication (**a**), transcription (**b**), and telomeres elongation (**c**).

More specifically, the biological roles of G4 can be unveiled by taking into account some main cellular events like replication, transcription, and telomeres elongation [30]; for instance, it has been observed that replication forks slow down when progressing through G-rich strand templates, in contrast to neighboring regions that lack such G-rich sequences and the presence of these non-canonical structures interrupts the normal catalytic activity of DNA polymerase leading to its stalling (Figure 2a) [31,32]. This is even more highlighted by the fact that prokaryotic and eukaryotic cells are equipped with a robust system of specialized helicases that effectively unfold G4s during DNA replication, allowing an effective fork progression, such as Pif1 in *S. cerevisiae* [33,34] and Bloom helicase (BLM) in mammalian cells [35,36]. Similarly, during DNA transcription, the presence of G4 on the DNA template strand blocks the movement of RNA polymerase, leading to the arrest of transcription, and G4 on non-template DNA strands is able to block the transcription process by forming an unusually stable RNA/DNA hybrid with the nascent RNA (Figure 2b) [36]. At the telomeric level, human DNA contains a double-stranded repeat region that has approximately 300–8000 precise CCCTAA/TTAGGG repeats, as well as the 3′-tail single-stranded repeat region with 10–50 TTAGGG repeats, which can form G4 structures, thereby regulating telomere metabolism [37,38,39]. G4s in telomeres can affect telomerase activity whose activation and upregulation are common in various cancers, indicating that strategies targeting the catalytic activity of this enzyme could have significant antiproliferative effects [40]. Furthermore, the promoter of the gene encoding the catalytic subunit of telomerase hTERT shows G-quadruplexes often used as targets for telomerase inhibition (Figure 2c) [41,42].

The presence of G4 at the telomeric level can also be discussed not only concerning DNA but for RNA as well. TERRA (Telomeric Repeat-containing RNAs) are non-coding RNAs that are the result of the transcription of telomeres in mammalian cells showing G-rich sequences, UUAGGG, that can fold into G4 structures [43,44,45]. Their role is yet to be fully clarified, but diverse hypotheses suggested that they might regulate telomerase activity and chromatin remodeling [46,47,48].

**Figure 3:**
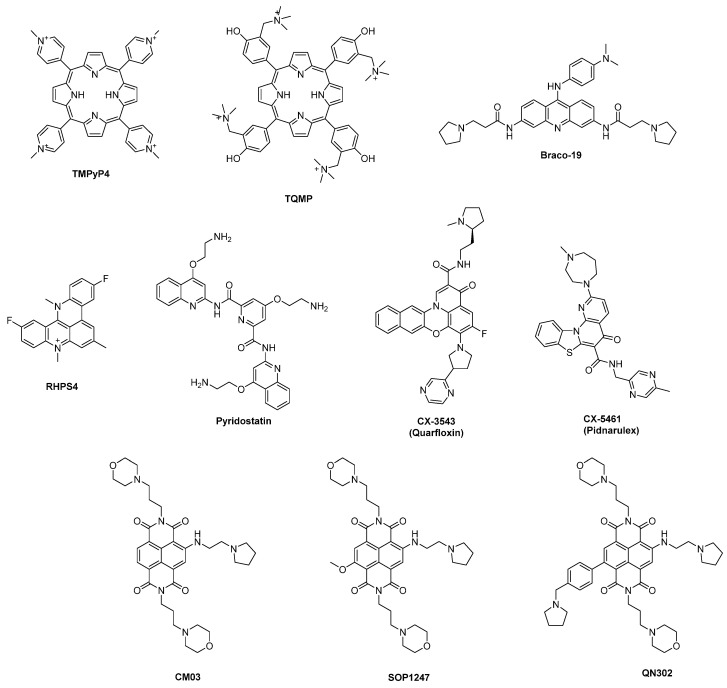
Chemical structures of some known G4 ligands.

After the discovery, the characterization and the investigation of the biological roles of G4 structures efforts were put to use them as innovative targets for pharmacological purposes through the design of molecules with mechanisms of action-based interactions with the sequences. Throughout the years, the most classical and traditional use of G4-binding molecules has been represented by stabilizers, e.g., chemical entities with recurring structural features that can be used to recognize the target and extend its presence within the genome overtime. The development of small molecules with binding and stabilization activity on G4 can be described following the timeline that led to the introduction of new potential candidates from 1997 to today [49]. The first relevant group is the one containing ligands bearing a porphyrin core. Among them **TMPyP4** was studied for its inhibition activity on telomerase and down-regulation of oncogenes like *c-MYC*, KRAS, and BCL-2 [50], but although **TMPyP4** displays high affinity for G-quadruplexes, it exhibits poor selectivity over duplex DNA [51]; to overcome this limitation, analog **TQMP** was synthetized, and obtained through the insertion of a phenolic ring inside the structure [52]. Furthermore, **BRACO-19**, a 3,6,9-trisubstituted acridine compound, is another important G4 ligand with inhibition activity of telomerase [53]. Its structure was expanded, leading to **RHPS4**, a pentacyclic system with a condensed aromatic center and no side chains. It exhibits remarkable telomerase inhibitory activity and can effectively inhibit or reduce the growth of cancer stem cells when combined with Paclitaxel [54]. Again, off-target toxicity and failed pre-clinical evaluations pushed for the development of a more selective compound like **Pyridostatin** (**PDS**), bearing a different aromatic arrangement without the presence of the polycyclic planar moieties that were responsible for non-specific intercalations through duplex DNA (Figure 3).

Although the described G4 ligands showed interesting outcomes during biophysical and in vitro assays, none of them reached advanced phases of development. In 2009, a new fluoroquinolone analog **CX-3543** (**Quarfloxin**) was a first-in-class G4-interacting drug that entered clinical trials, reaching phase II for the treatment of neuro-endocrine carcinomas [55], with effects on RNA polymerase I, inhibiting its transcription activity and triggering apoptosis in cancer cells. Despite these features, Quarfloxin was eventually withdrawn from clinical studies because of the lack of efficacy and bioavailability problems. A structure optimization process led to **CX-5461** (**Pidnarulex**) which also reached phase I of clinical trials thanks to its activity on RNA polymerase I. However, problems regarding partial inefficacy, phototoxicity, and mutagenic issues came out, stopping its development process [56,57]. The naphthalenediimide (NDI) core was also optimized to gain new hits like **CM03**, **SOP1247**, and **QN-302**, which differ from the side chains used to functionalize the central core (Figure 3). The latter showed the most promising results in vitro with a vast plethora of down-regulated genes and after its involvement in further studies in 2023, it resulted in being the most advanced ligand in clinical trials among G4 ligands [58].

The limited availability of clinically effective ligands pushed, on one hand, for the synthesis of new potential candidates and on the other hand, for deeper insights into the challenges of selective targeting. Since the knowledge about G4 is still far from being completely unraveled and many aspects remain to be fully elucidated, new resolutions are needed. An increasing number of G4-stabilizing molecules are known today (>1000) [17]; however, the chemistry implied to design new analogs did not lead to the production of small molecules only, but more complex and sophisticated systems have been studied through the years. As a consequence, beyond the more traditional involvement of G4 as targets to gain pharmacological effects, e.g., antiproliferative outcomes, new strategies are being researched, not to directly observe changes at the cellular level, but also to obtain new genome information, together with mechanistic explorations, and support the development of new diagnostic strategies also through the visualization of G4. Although some so-called “imperfect G4s” (imG4s) are known—such as G-quadruplexes with missing or interrupted tetrads, bulges (buG4s), or irregular strand orientations [59,60] and strategies for their detection are emerging [61,62]—this review will focus on classical G4s, highlighting the latest approaches developed to study, detect, and manipulate G4 structures both in vitro and in vivo. With this purpose, we can categorize the molecular tools into three main classes: fluorescent probes, metal complexes, and conjugates.

## 2. Small-Molecules Fluorescent Probes for G4 Live Imaging

The discovery of G4 ligands paved the way to more innovative systems where the binding activity of these compounds was linked to light-responsive behaviors, gaining live imaging of G4s and their detection in live cells through the application of fluorescent probes. Ideally, these systems need to present precise features that will make them useful for their scope; apart from specificity, selectivity, and cell permeability, which are typical features for traditional ligands, they need to present turn-on fluorescence behavior, a high quantum yield, and high photostability [63]. Furthermore, they generally present a three-functional structure with a recognition unit for the interaction with non-canonical structures of the nucleic acid and the fluorophore, connected by a linker [64]. A first classification among fluorescent probes can be used to identify five types [11]: (i) “light-up” probes become more fluorescent upon binding the target, (ii) while “light-off” ones work contrarily, as they decrease in fluorescence when bound; (iii) probes could also have a permanent fluorescence upon G4 interaction or (iv) have different emission maxima between free, and (v) bound state. Moreover, probes may not only be pivotal in G4 morphology and ligand-interaction studies but can also act as theranostic agents combining therapeutics and diagnostics for image-guided therapy [65,66]. When probes are used, it is crucial to select irradiation wavelengths that can match the probes’ properties to avoid first of all tissue autofluorescence range at 300–700 nm. Near-infrared (NIR) wavelengths are often preferred and typically, compounds with large Stokes shift are the most common tools since they present a high difference between the emission and the excitation wavelengths, making them more useful to improve signal clarity and detection accuracy. Fluorescent G4-selective probes can be categorized through a classification based on their structural features. The main families are carbazoles, benzothiazoles, polyphenyl derivatives, guanine-containing probes, porphyrins, and phthalocyanines.

### 2.1. Carbazoles

**Figure 4:**
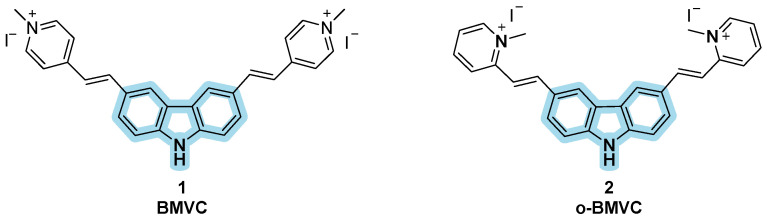
Structures of BMVC (**1**) and o-BMVC (**2**) fluorescent probes bearing a carbazole moiety.

Carbazole is a tricyclic aromatic compound consisting of two benzene rings fused on either side of a five-membered nitrogen-containing moiety [67]. Due to its extensive electron delocalization, carbazole has been largely applied as fluorescent probe, since it posses positive features such as large Stoke shift and high quantum yield, as well as biocompatibility and good stability [68]. Compound 3,6-bis(1-methyl-4-vinylpyridinium)carbazolediiodide (**BMVC**) (Figure 4, compound **1**) was reported in the early 2000s by Chang et al. as a sensitive G4 DNA ligand, and it was among the earlier fluorescent probes applied for the in vivo imaging of G4 structures, and was able to bind the quadruplex in the human telomeric sequence d(T2AG3)4 [69]. It is a “light-up” probe since its fluorescence increases upon binding and presents a large time of adhesion [70]. The compound was used in a plethora of models on cancer-induced cells to see if it could be useful for early cancer detection by the evaluation of its fluorescence in neoplastic models through fluorescence microscopy: after the induction of cancer in mouse fibroblast cell lines BALB/c 3T3, transformed cells were later treated with **BMVC** to detect tipycal cancer cells behavior such as proliferation, increased motility, and invasiveness. Interpretation of the fluorescence signals confirmed the typical neoplastic phenotype and all these results made **BMVC** a valid marker for cancer detection with particular attention on its selective modality of action [71]. The effects of **BMVC** in neoplastic cells and not in healthy ones is indeed linked to its permanence in lysosomes: using specific dyes, it was possible to observe the prolonged retention of **BMVC** in lysosomes of normal cells with no interaction with the nucleic acid confined into the cellular nucleus. In neoplastic cells, the modified features of the diverse compartments can help the compound to escape from the lysosomes and penetrate into the nucleus where fluorescence is enhanced after G4 interaction. Probe activity at the nuclear level is also confirmed by studies of the co-presence of nuclease enzymes that reduce the fluorescence signal. All these findings have helped **BMVC** to be considered as a useful cancer diagnostic tool; however, some relevant drawbacks have emerged during experimentations, such as low selectivity on the duplex at high concentrations, and non-ideal properties like short Stokes shift, and photobleaching, making its application in cellular and vivo more complicated [72]. To overcome these issues, structural modifications were made, leading to isomer **o-BMVC** by Tseng et al., obtained through changings in the position of the vinylpyridinium substituents of the probe (Figure 4, compound **2**). The group used time-gated Fluorescence Lifetime Imaging Microscopy (FLIM) to measure the fluorescence decay time of this probe upon interaction with several DNA sequences, as well as to reveal its distribution in the cell. Image identification of **o-BMVC** foci—e.g., clusters of G4 bound to the probes—was possible through staining HeLa cancer cells and MRC-5 normal cells. Furthermore, the use of **o-BMVC** foci as biomarker for cancer clinical screening was tested through comparison of time-gated FLIM binary images of oral samples collected from healthy volunteers and of head and neck cancer (HNC) samples. The results showed a higher number of **o-BMVC** foci in tumors than in normal cells and an incremented number of foci at advanced cancer stages, hinting to a possible correlation between tumor burden and disease stage. In fact, the study suggests the use of this probe as a potential clinical test for screening oral cancer through minimally invasive oral swabs [73,74].

### 2.2. Benzothiazoles

**Figure 5:**
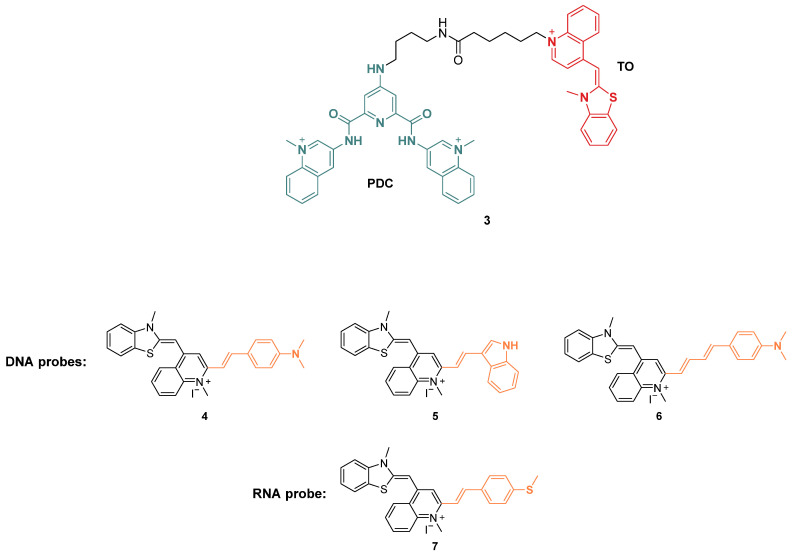
Structures of fluorescent probes bearing a benzothiazole moiety. Conjugation of TO to PDC (**3**) and functionalization with styryl derivatives leading to probes used for G4 DNA (**4**–**6**) and G4 RNA detection (**7**).

Benzothiazole consists of a benzene ring fused to a five-membered thiazole ring. This moiety has been inserted in compounds with different pharmacological activity such as antibacterial [75], antiviral [76], and antitumoral compounds [77]; however, benzothiazole derivatives are good fluorescent probes for G4 identification as well. In this context, their use is related to one of the oldest cyanine dyes, **thiazole orange** (**TO**), where the benzothiazole unit is connected to a quinoline ring to afford this “light-up” probe [78], however selectivity problems emerged due to the ability of **TO** in interacting with duplex DNA, either through intercalation or interaction with the grooves. Structural modifications were needed to improve **TO** selectivity on the G-quadruplex to use it as a molecular tool for the detection of these structures and this led to two main changings. The first type of modification was performed by linking the structure of **TO** to known G4 ligands, while a second type of modification was strictly related to a change in its structure, changing the chemical moieties already present. From the first group, ligands like **pyridodicarboxamide** (**PDC**) [79] (Figure 5, compound **3**) and cryptolepine [80] were linked to **TO**, producing chimeric molecules with increased selectivity and fluorescence upon binding. Among the second type of modifications, Chow et al. led the design of **styryl-TO** derivatives with enhanced selectivity either towards DNA or RNA G4 whose fluorescence was studied in presence of DNase and RNase enzymes (Figure 5, compounds **4**–**7**) [81].

**Figure 6:**
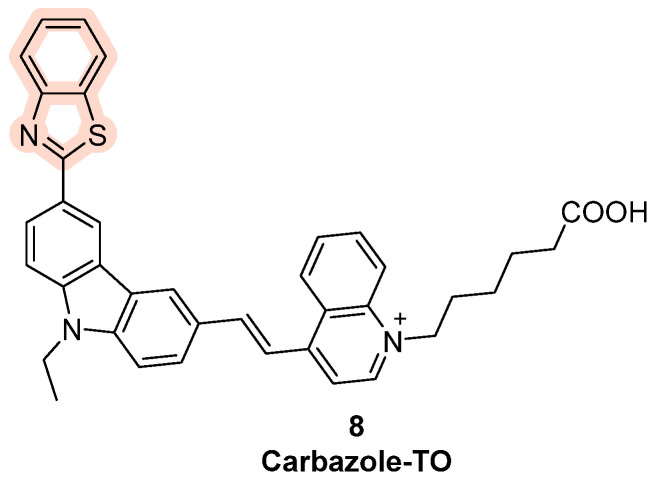
Structure of **Carbazole-TO** obtained through the chemical modification of thiazole orange with the insertion of a carbazole moiety.

On the other hand, upon structural changes on the structure of **TO**, Yang et al. obtained **Carbazole-TO** with the incorporation of a carbazole ring in the original scaffold (Figure 6, compound **8**); its fluorescence increases significantly when bound to G4 units in Bcl-2, while the binding to other G4s causes a minor increase in fluorescence, making **Carbazole**-TO a specific probe for this particular G4 sequence [72]. In general, new series of compounds based on the structure of **TO** have been synthesized in order to increase specificity and to obtain new effective fluorescence ligands too [82].

**Figure 7:**
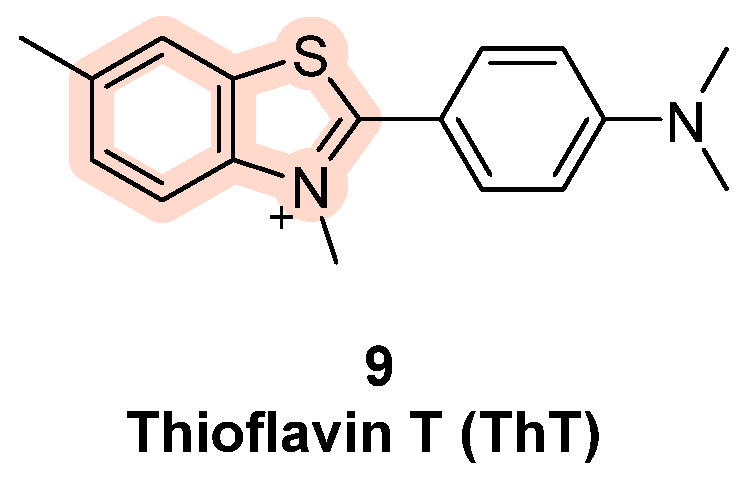
Structure of ThT (**9**). ThT exhibits strong fluorescence enhancement in the presence of G4 sequences while single- and double-stranded controls show less relevant effects. These results confirmed ThT as a specific probe for G4 structures.

Other probes have been designed with a benzothiazole moiety in their structure, such as **Thioflavin T** (**ThT**), which shows fluorescence upon binding with telomeric DNA (Figure 7, compound **9**). In order to test the probe, fluorescence emission spectra of ThT binding different oligonucleotides have been registered; compared to **ThT** alone its fluorescence intensity in the presence of G4 was much higher, while other double- and single-stranded DNA sequences caused no relevant increase in emission. The validation of this assay was performed on four G-rich aptamers of unknown secondary structure (Tet-7, Tet-15, Tet-19, and Tet-24): each one showed significant fluorescence enhancement with good FI/FI_0_ values, indicating the ratio between the measured fluorescence intensity and the intensity of **ThT** alone. The results led to the understanding that these sequences fold into G4 and that **ThT** is a specific probe for G4 investigation [83].

**Figure 8:**
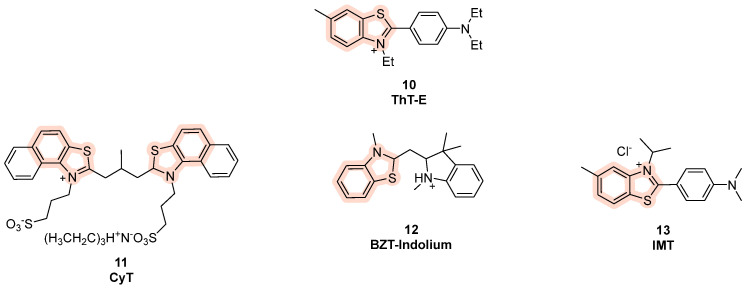
Structures of benzothiazole-based fluorescent probes for G4 detection.

The affinity and selectivity for the G4 structures further increases in **ThT-E**, obtained by Guan et al. from **ThT** by the subtitition of the three methyls with ethyl groups (Figure 8, compound **10**) [84]. Another cyanine dye, called **Cyt**, is able to recognize RNA G4 and is used for live imaging, with different fluorescent outcomes in different temperature conditions (Figure 8, compound **11**) [72], while compound **BZT-indolium**, was proved to stabilize the G4 in the promoter sequence of *c-MYC*, causing the downregulation of the oncogene expression (Figure 8, compound **12**) [72]. Finally, Tang et al. have studied the applicability of the benzothiazole derivative **IMT** for G4 visualization and observed that the probe co-localized with the G4-specific antibody BG4, reporting increase in fluorescent foci during the entire cell cycle, thus making **IMT** the best known probe to trace real-time changes in G4 structures in living cells (Figure 8, compound **13**) [85].

**Figure 9:**
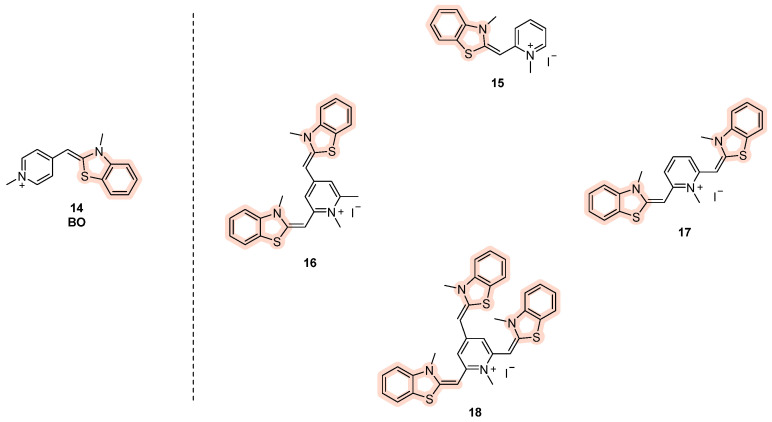
Structures of benzothiazole-based cyanine derived from **BO** (compound **14**), with mono- (**15**), bi- (**16**,**17**), and tri- (**18**) substituted analogs.

Among benzothiazole-based cyanine fluorescent “light-up” probes, benzothiazolylmethylidene pyridinium derivatives (Figure 9, compound **14**) were also described by Turaev et al. [86]. A series of mono-, bi-, and tri-substituted analogs were indeed developed, and one of them (Figure 9, compound **16**) emerged as promising, with levels of emitted light upon excitation comparable with **thioflavin T**, showing selectivity towards parallel and hybrid G4s rather duplex DNA.

### 2.3. Polyphenyl Derivatives

**Figure 10:**
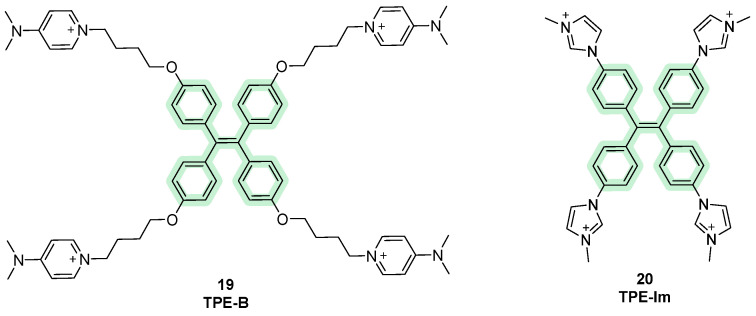
Structures of fluorescent probes bearing the tetraphenylethene moiety.

Among recent and active aromatic cores, tetraphenylethene (TPE) has been one of the most studied for its positive fluorescence properties and geometry features that allow for the recognition of telomeric G4s [87]. TPE consists of a conjugated system composed by four phenyl rings linked to eachother by an ethyelene spacer, bearing a non-planar rearrangemnent due to the steryc hindrance between adjacent aromatic groups. This spatial disposition makes the molecules non-emissive in solutions because after irradiation, the energy is not released through fluorescence but dissipated in non-riadiative forms, such as rotational. On the other hand, when they are found in a solid state, they can pack and aggregate, making the emission of radiation through fluorescence easier since rotation is restricted. This photophysical property is called aggregation-induced emission (AIE) and is rapresentative of TPE probes, as in the cellular context, the interactions with the target block the rotational movements and lead to the observed fluorescence [88,89]. Scarce water solubility and selectivity led to the design of some derivatives like **TPE-B** (Figure 10, compound **19**), selectively probing parallel topologies of G4 with 1:1 stoichiometry. TPE-B binds to the end-stacking sites of the quadruplex with the main core interacting through π-π stacking and the side chains interacting with the grooves. Also in this case, upon binding, intramolecular rotations are restricted to generating AIEs [90]. Another derivative, **TPE-Im**, was obtained by the attachment of four imidazolium moieties to the TPE core (Figure 10, compound **20**). The electrostatic interactions created by imidazolium chains with the DNA backbone adds to the π-π interactions given by the four conjugated benzenes, making **TPE-Im** a well-suited G4 probe. More specifically, interactions with Tel22 sequences and turn-on fluorescence emissions were observed [91].

**Figure 11:**
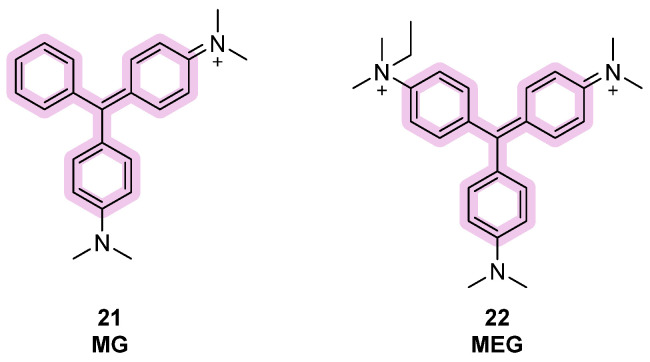
Structures of fluorescent probes bearing the triphenylmethane moiety.

Apart from TPE dyes, **triphenylmethane** (**TPM**) dyes can also be considered among useful molecular tools for G4 detection. In this case, only three phenyl substituents are present in the structure and AIE phenomena are observed as well. Studies have shown binding activity on intramolecular G4 rather than intermolecular conformations, with **malachite green** (**MG**), introduced in 2007, being one of the first compounds belonging to this class (Figure 11, compound **21**). During the following years, other analogs were designed, keeping the same central scaffold but changing the amino substituents on it, with **methyl green** (**MEG**) being the most recent (Figure 11, compound **22**). The binding stoichiometry observed was 2:1, although an increased fluorescence was observed as well as the presence of the duplex, indicating selectivity problems [92].

**Figure 12:**
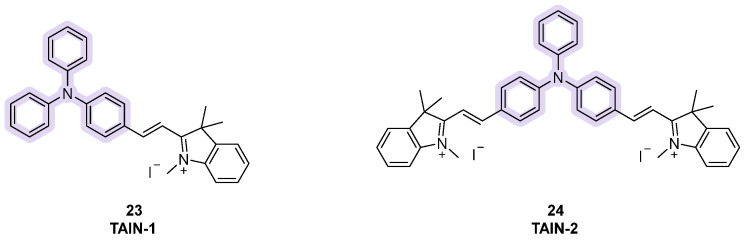
Structures of fluorescent probes bearing the triphenylamine moiety.

A third class of fluorescent dyes for G4 detection is the one cointaing **triphenylamine** (**TPA**), where three phenyl rings are linked to each other through a central tertiary amine. The scaffold has been decorated by inserting some electro-withdrawing and positvely charged indolium moieties into the structure, leading to diverse compounds able to perform AIEs at near-infrared wavelengths and consequently they become useful probes. Among them, **TAIN-1** and **TAIN-2** were designed and synthesized by changing the number of side chains on the aromatic core, and later used for a set of experiments by Ming-Hao Hu et al. (Figure 12, compounds **23**, **24**). When in the presence of G-quadruplex-forming sequences, fluorescence spectra were recorded and for both compounds, enhanced emissions were observed with maxima around 740 nm. However, a higher fluorescence enhancement was observed for **TAIN-2**, as well as a stronger stabilization effect on G4, a longer fluorescence signal, and a longer absortion wavelength in the visible region (555 nm). Consequently, **TAIN-2** was selected for advanced studies and more information on its modality of action was gained. For instance, a binding profile with a 1:1 stoichiometry was determined [93]. In competition with **PDS**, a lowered fluorescence signal confirmed a similar binding modality, based on interactions with the terminal G-quartet of the sequence [94]. *In cellulo* studies showed localization of **TAIN-2** in the cytoplasm and the deletion of radiative emissions in the presence of DNase, which led to the hypothesis of a binding from **TAIN-2** in the mythocondrial DNA present in the cytoplasmatic area of the cell. In live cells, studies with DMS, a staining agent used to prevent G4 formation, saw a decrease in the number of fluorescence foci, which confirmed the selectivity of **TAIN-2** to G4 [93].

### 2.4. G-Clamp Derivatives

**Figure 13:**
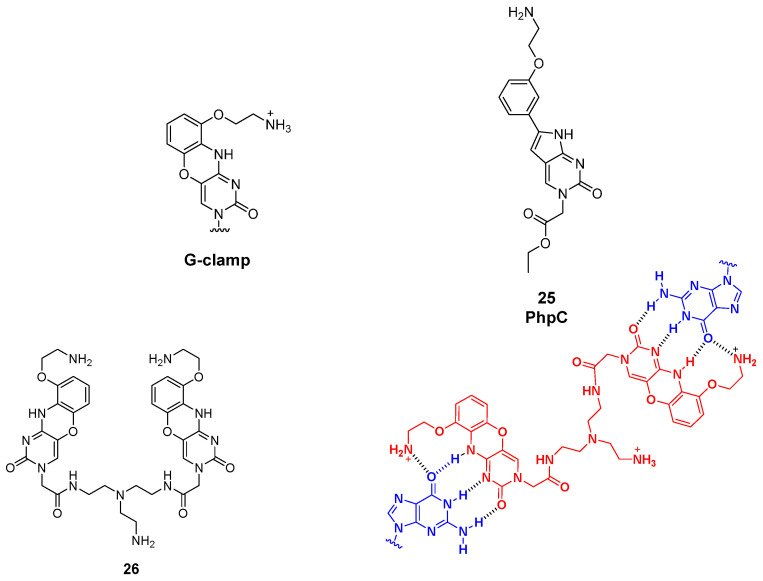
General G-clamp structure and **PhpC** derivative (**25**). Structure of the dimeric G-clamp analog (**26**) and its interaction with guanine residues in target RNA G-rich sequences.

G-clamp are phenoxazine derivatives known for their interactions with nucleic acids. More specifically, they are able to interact with G-rich sequences through formations of H-bonds with guanine residues. Different analogs are known today, such as phenylpyrrolocytosine (**PhpC**), known for its disrupting effects on G4. **PhpC** is a fluorescent G-clamp analog that can “trap” guanine bases that transiently flip out of a G-quadruplex (G4) structure [95]. When the G4 is stable (e.g., in high K^+^ conditions), the external guanines remain stacked and **PhpC** fluorescence is relatively high. If G4 stability decreases (e.g., low K^+^ conditions), guanines at the external tetrads can transiently flip out, **PhpC** intercalates or binds these flipped-out guanines, and its fluorescence decreases. Therefore, **PhpC** serves as a sensitive fluorescent reporter for G4 dynamics and stability, particularly capturing transient structural fluctuations [95]. Also, some G-clamp dimers derivatives were described [96]. In this case, dimer **26** (Figure 13) is a specialized ligand that can recognize two guanines in close proximity. In the context of RNA G-quadruplexes, such as r(CGG)_n_ repeats, the dimer binds the stem region and promotes a structural transition from the G4 conformation to a hairpin. Experimentally, this can be monitored using **ThT** fluorescence: the addition of the dimer causes a concentration-dependent drop in **ThT** signal because **ThT** preferentially binds G4s. Therefore, the G-clamp dimer can act as a molecular tool or “probe” to detect and modulate RNA G4 structures, reporting on G4 presence and dynamics via fluorescence readouts.

### 2.5. Guanine-Containing Probes

**Figure 14:**
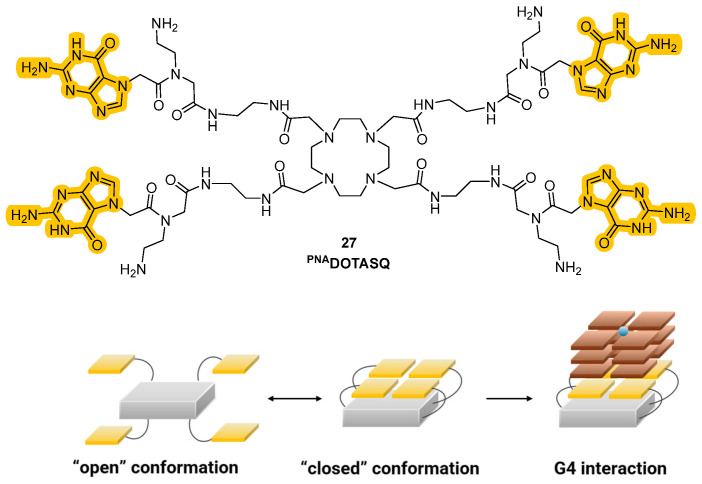
Structure of a “smart” G4 ligand showing the presence of guanine residues in the side arms (**up**). In proximity to G4, interactions between the residues will lead to the formation of intramolecular G-tetrads and to target interactions (**down**).

In 2015, an alternative strategy to classical turn-on probes was proposed by Monchaud et al., who followed the idea of merging the binding activity of ligands with their properties of fluorescence. The so-called “twice-as-smart” ligands are structurally dynamic molecules that are able to self-assemble in quartets when interacting with the G4, making them smart G4 ligands and, at the same time, smart fluorescent probes, as their fluorescence is turned on upon interaction with the target [97]. These ligands represent the evolved and more sophisticated form of “smart ligands” including the first prototype **^PNA^DOTASQ** (Figure 14, compound **27**). The structural features of these compounds are based on the presence of an aromatic planar core that is usually found in most of the known G4 ligands, and of four guanine-enriched chains, inserted through studied synthetic routes [98]. The “smart” adjective is linked to their ability in recognizing G-quartets and in their proximity, being involved in intramolecular interactions that will lead to the formation of intramolecular G tetrads starting the so-called “like-likes-like” quarter/quartet interactions with the target G4.

**Figure 15:**
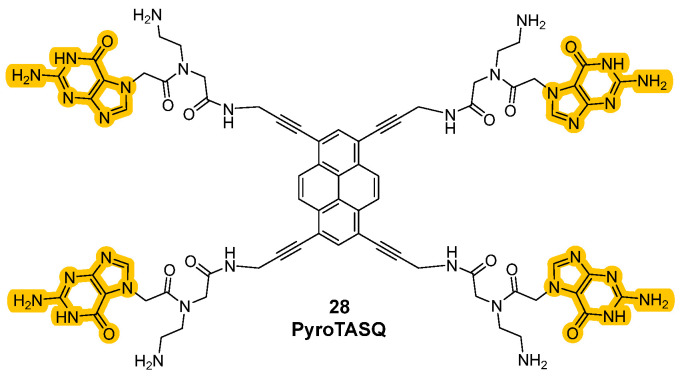

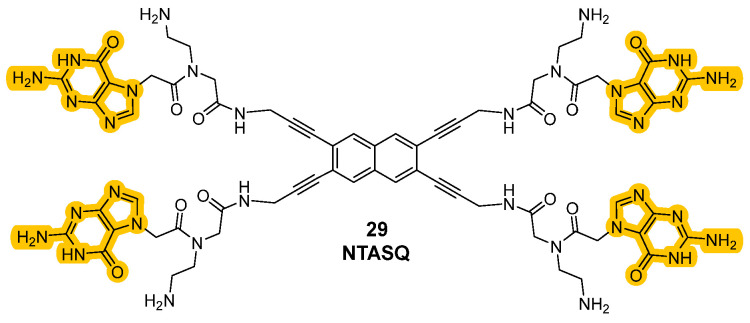
Structures of “twice-as-smart” G4 ligands PyroTASQ (**28**) and NTASQ (**29**).

Historically, the introduction of these analogs started from the older class of biomimetic ligands that represented the first G4 stabilization approaches based on structure similarities. The passage from “smart ligands” to “twice-as-smart ligands” was achieved by giving these molecules the features of fluorescent probes, allowing the direct visualization of quadruplexes in cells. The two main structural elements previously described are crucial for the modality of action, since the interaction between the core and the chains correlates with the mechanism of the ligands and their emission properties. Specifically, when the compounds are not in the presence of G-quartets they can be found in a defined “open” conformation where the four side chains are not interacting with each other. In this form, when the aromatic core is excited, the energy is transferred to the electron-rich guanine chains and instead of observing relaxion and fluorescence emission, the energy is converted through other pathways, hence stimulating photoinduced electron transfer (PET) that will not show fluorescence emission. This happens in the “open” conformation because the side chains and the central core are also involved in orbital overlap that favors charge distribution. A conformational change can be observed upon interaction with the G4-forming regions of the nucleic acids that induce the intramolecular interactions between the chains and the formation of intramolecular G-tetrad. This leads to a more rigid system, where the electronic transfer from the aromatic core to the side chains is not possible anymore, so that after excitation and relaxation, the energy is converted into fluorescence, which is then observed. The phenomena that decrease and shut down fluorescence in the “open” form are not present in the “closed” one and the interconversion of one form into another expereinces a “semi-closed” form which was studied to better clarify the transfers of energy (Figure 14) [98,99,100]. Synthetic efforts to afford these smart ligands known as the Template-Assembled Synthetic G-Quartet (**TASQ**) produced highly differentiated compounds, obtained using different aromatic central cores. Among the synthetized derivatives, **PyroTASQ** (Figure 15, compound **28**), bearing a pyrene core was the first, but it showed limiting behaviors such as extracellular aggregation and a consequent weak G4 interaction [101]. To overcome this problem, the hindrance of the aromatic part was reduced by designing **Naphto-TASQ** (**N-TASQ**), where the pyrene was substituted with a naphthalene core (Figure 15, compound **29**). In this way, aggregation was reduced, bioavailability was enhanced and low toxicity (IC_50_ > 100 μM after 72 h treatment) was observed [98]. Furthermore, the new ligand also showed good outcomes such as more selectivity and efficacy in the interaction with G4-forming sequences like *c-MYC* and TERRA.

**Figure 16:**
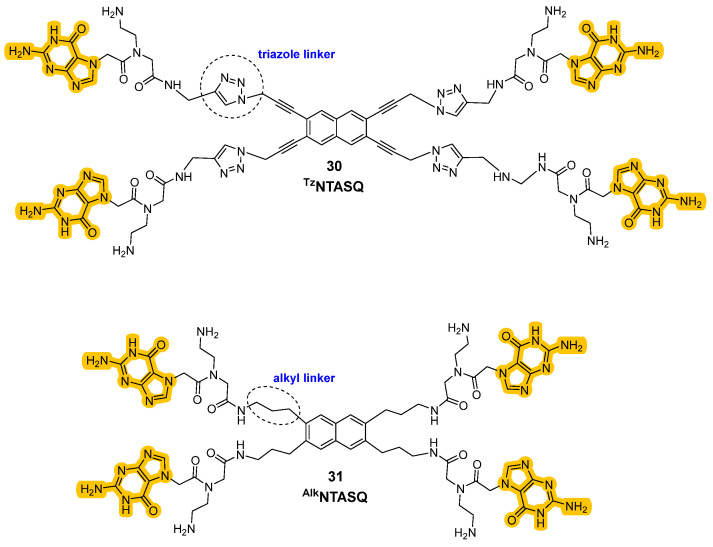
Structures of new “twice-as-smart” ligands where the nature of the linker between the core and the guanines side chains was changed by functionalization with a triazole moiety (**30**) and a propylamine chain (**31**).

The novelty of the **N-TASQ** probe is not related to its peculiar structure and mechanism of interaction only, but to its uncommon spectroscopic properties as well. Contrarily to other probes, it presents a low absorbance maximum (320 nm), making the recording of images more difficult due to problems of tissue autoflorescence that can impact the results. This low value of maximum absorbance wavelengths would indeed request UV light for excitation, which can be problematic for the live-imaging purposes. However, the interactions between the probe, G4, and restricted water molecules create a tight environment that changes the relaxation of the probe after excitation, as decribed by the Red Edge Effect (REE) [102]. More specifically, even low-energy (redder) light can excite the probe, and thanks to the REE higher wavelengths can be used for the purposes making two-photon (720 nm) and confocal microscopy (408, 488, 555 nm) useful to achieve clearer and sharper images of G4 with less interference. All these phenomena are more enhanced in slow-dynamic systems such as fixed cells, but they are also compatible with live cells. For all these reasons, **N-TASQ** was used in both fixed cells, with confocal microscopy, and in live cells using two-photon microscopy to minimize cellular stress. Preferred interactions with G4s and RNA were observed, as confirmed by the colocalization of **N-TASQ** with the G4-binding antibody BG4. Moreover, through live-cell imaging systems, the time required for cell penetration (12 h) and its permanence inside the cellular environment (100 h) were recorded. The probe was also used to record G4 presence in a plethora of nervous cellular phenotypes, demonstrating its versatility [102]. More recent studies have led to the synthesis of two additional TASQ derivatives, **^Tz^N-TASQ** and **^Alk^N-TASQ**, where the conjugated alkyne present in the linkers between naphthalene and guanines in the original probe were differentiated (Figure 16, compounds **30**, **31**). In **^Tz^N-TASQ** the amidic bond was replaced by a triazole, which preserves conjugation and increases the rigidity of the probe, while in **^Alk^N-TASQ,** a reduction reaction was performed on the alkyne to a propylamine linker, disrupting the conjugation and increasing the flexibility. The study provided important information on the rational design of TASQs; a less-flexible structure is preferred, as it increases both the affinity and the efficiency of the “open–closed” interconversion [99]. N-TASQ probes proved to be particularly useful for the evaluation of the modality of interaction of other ligands to G4. When co-incubated with potential G4 ligands, a rise in N-TASQ fluorescence signifies ligand-induced stabilization of the G4, while decreased or unchanged fluorescence suggests destabilization or absent interaction. This allowed researchers to visually and spectroscopically confirm whether a molecule preserves, destabilizes, or does not interact with a G4 structure [103].

### 2.6. Porphyrines and Phthalocyanines

**Figure 17:**
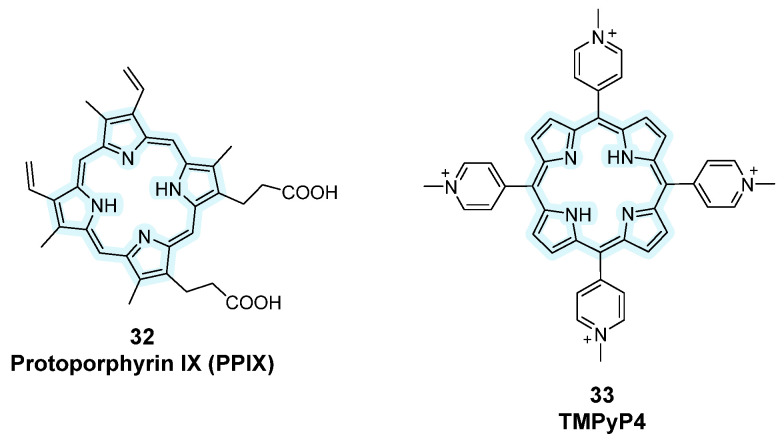
Structures of fluorescent probes bearing the porphyrin moiety.

This class of compounds is characterized by a macromolecular heterocyclic structure containing four pyrrole subunits interconnected via methine bridges. Due to the presence of a 26 π-electron highly conjugated core, porphyrins possess distinctive optical properties and fluorescence [104]. These macrocycles can be properly modified, and the derivatives seem to be able to target G-quadruplexes via π-π stacking interactions, but these compounds were mainly studied for their effects on cancer cells rather than as fluorescent G4 probes. The periphery of the planar core can be substituted with negatively charged carboxylic acid groups, like in **Protoporphyrin IX** (**PPIX**), a selective ligand parallel to G4 (Figure 17, compound **32**). However, more common substituents are positively charged groups, like quaternary piridinium at the meso position, which can be found in **TMPyP4** (Figure 17, compound **33**). This cationic porphyrin was found to inhibit tumor growth through the downregulation of *c-MYC* and human telomerase reverse transcriptase but its non-selectivity and consequent toxicity have stopped its development as a ligand as well.

**Figure 18:**
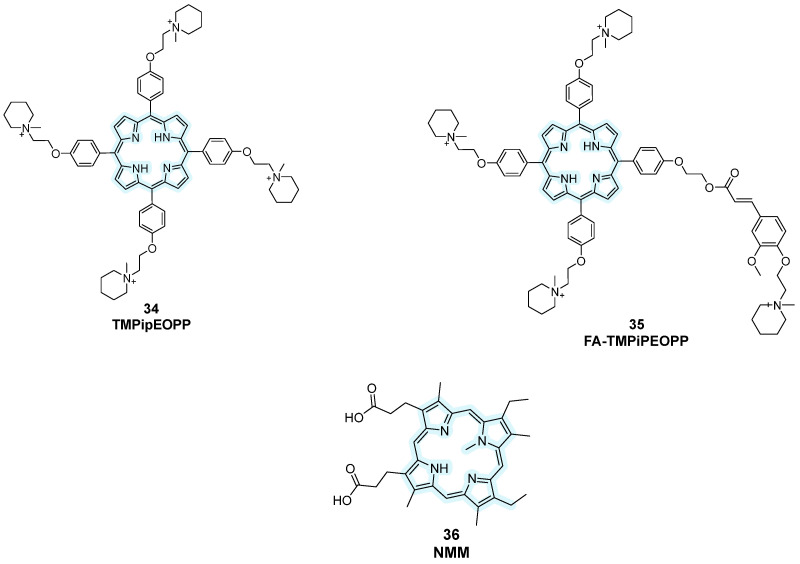
Structures of more recent porphyrin-containing fluorescent probes. Structural changes were performed starting from previous analogs bearing the same central core to optimize their activity.

Later on, Zhang et al. designed **TMPipEOPP** by elongating the cationic moieties with bulkier side arms, reducing intercalation with duplex DNA and improving G4 selectivity (Figure 18, compound **34**) [105]. It was revealed that this compound is particularly interesting as a human telomere-targeting photosensitizer for photodynamic cancer therapy. However, its incompatibility with acidic conditions required new derivatives and that is why a further functionalization by the same research group with ferulic acid (FA) was performed, leading to **FA-TMPipEOPP**, a water-soluble, cationic, porphyirinic derivative that is less prone to protonation, making this compound useful as a probe for G4 recognition in acidic enviornments (Figure 18, compound **35**) [106]. **N-methyl mesoporphyrin IX** (**NMM**) also showed high selectivity for G4 parallel conformations via end-stacking with weaker interactions towards antiparallel structures, and fluorescence enhancement upon binding, finding its application in biochemical assays and fluorescence imaging (Figure 18, compound **36**) [107].

**Figure 19:**
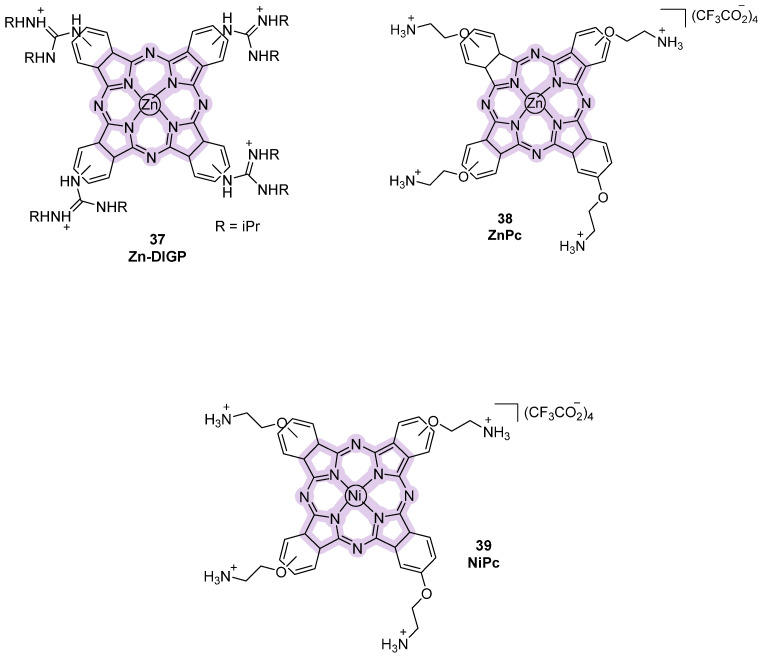
Structures of phthalocyanine-derivative fluorescent probes.

Another class of probes containing pyrrole subunits is represented by phthalocyanines, consisting of four isoindole groups linked to each other by nitrogen atoms, hence showing a wider planar surface for electron delocalization [108]. Among them, **Zn-DIGP** bearing a zinc (II) cation in its structure was studied and properities of the theranostic fluorescent probe were observed with a down-regulation of *c-MYC* and KRAS expression in cancer lines (Figure 19, compound **37**) [109,110]. Starting from this analog, novel metallo-phtalocyanines (MPcs) with G4-stabilizing activity were rationally designed like **ZnPc-** and **NiPc-** bearing zinc (II) and nickel (II) into the phtalocyanine macrocycle (Figure 19, compounds **38**, **39**). Their ability to bind and stabilize G4 over duplex DNA was confirmed through FRET melting and other biophysical assays, marking a significant step forward in the design of multi-functional G4-targeting agents [110].

### 2.7. Nucleoside-Derivative Fluorescent Small Molecules

Nucleoside derivatives have emerged as powerful tools for probing G4 structures due to their ability to be site-specifically incorporated into DNA sequences and report on local conformational changes. These derivatives often offer higher selectivity for specific G4 topologies and improved features compared to other small-molecule probes, allowing them to be used effectively for detecting and monitoring G4 folding and dynamics. Thanks to their advantages, they can be considered more useful than other strategies that only provide generic information on the formation of non-canonical structures [111]. In these strategies, the synthesized fluorescent nucleosides in their phosphoramidite forms are incorporated into oligonucleotide sequences, typically replacing residues in positions that do not significantly affect G4 formation, generating covalently attached probes. After confirming that the substitution did not perturb the native G4 topology, various analyses can be performed. Typically, sequences capable of forming multiple G4 topologies (parallel, antiparallel, hybrids) are chosen, so that by changing the position of the modified nucleoside within the sequence and observing the resulting fluorescence, information about the specific topology can be obtained. Indeed, position-dependent changes in fluorescence emissions provide insights into G4 folding, offering a site-specific technique that can be used for structural studies and diagnostic applications.

**Figure 20:**
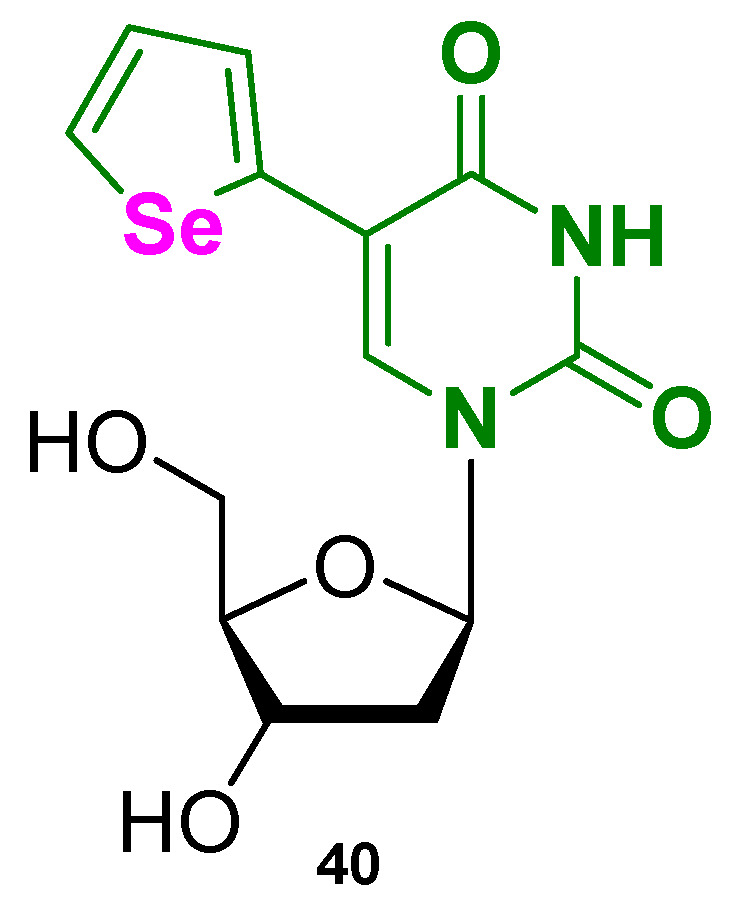
Structure of the nucleoside-based fluorescent small molecule ^Se^dU (**40**).

One example is the selenium (Se)-containing uridine analog, **^Se^dU** (Figure 20, compound **40**), which was obtained by incorporation at position 5 of uridine of a furane ring where oxygen was substituted with selenium to create a fluorescent nucleoside probe [112]. **^Se^dU** was inserted into the loop regions of different G4-forming telomeric sequences (H-telo), replacing thymine. By using various ionic conditions (i.e., Na^+^ to favor antiparallel, K^+^ for hybrid forms, and Sr^2+^ for parallel topologies) different oligonucleotides (ONs), each with the probe at different loop positions, were characterized. One specific ON, with the probe at T11 in the second loop of the sequence, showed the highest sensitivity, meaning it produced the clearest and most distinguishable fluorescence changes for different topologies. This position-dependent responsiveness allowed the modified ON to be selected for further analysis, enabling not only the sensitive detection of G4 formation and topology, but also the assessment of ligand-binding behavior, as changes in fluorescence directly reported interactions at that specific site. The sequences were indeed titrated with ligands under the conditions that favor different topologies, using well-known binders such as **PDS** and **BRACO-19**. When a fluorescent probe is found nearby the interaction site of the ligand, the binding can quench the fluorescence through π–π stacking or electron/energy transfer, resulting in a decreased emission. With **^Se^dU**, this decrease was used to study ligand preference for a specific topology. For example, with a modified ON prepared under conditions favoring the parallel G4, when a ligand like PDS was added, a significant decrease in fluorescence indicated that the ligand binds preferentially to the parallel form. Conversely, if the ligand does not prefer that topology, the probe’s environment remains largely unchanged, and little or no fluorescence decrease is observed. This approach demonstrates the utility of **^Se^dU** in studying ligand-binding specificity and topology preference in G4 structures. Furthermore, the selenium atom in the **^Se^dU** probe provided an additional advantage for X-ray crystallography. Selenium serves as an anomalous scatterer, enabling phase determination through the SAD (Single-wavelength Anomalous Dispersion) method, which allows the high-resolution structure determination of G4s. The **^Se^dU**-labeled oligonucleotides therefore not only functioned as fluorescent reporters in solution but also facilitated the crystallographic analysis of G4 structures. This dual functionality demonstrated the utility of the probe as a diagnostic and structural tool, providing atomic-level insights into G4 folding and enabling the design of structure-specific ligands or binders.

**Figure 21:**
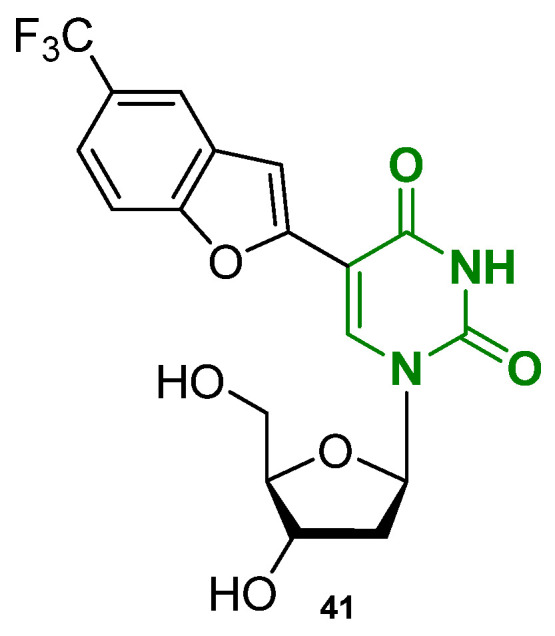
Structure of the nucleoside-based fluorescent small molecule TFBF-dU (**41**).

Another work based on modified nucleosides for G4 probing was produced by Khatik et al. that designed some fluorobenzofuran-modified nucleosides, including the most promising **TFBF-dU**, bearing a trifluoromethyl-benzofuran inserted at position 5 of uridine (Figure 21, compound **41**) [113]. Using **TFBF-dU**, the authors probed the cancer-involved EGFR promoter that bears a G4-forming sequence. Similarly to the previous work, the probe differentiated conformations under diverse ionic and ligand-binding conditions but two novelties can be identified. The first concerns the incorporation of fluorine atoms into the nucleoside probe, enabling the use of ^19^F NMR spectroscopy. Conventional ^1^H NMR is often insufficient to fully resolve G4 topologies, as overlapping signals make it difficult to distinguish different conformations. In contrast, the ^19^F-labeled nucleoside provides distinct, well-resolved signals that are exclusively associated with the probe, minimizing the risk of misinterpretation. In a picke dmodified ON, ^1^H NMR showed broad imino proton peaks (10–12 ppm), indicating multiple G4 topologies in equilibrium, but could not clearly track topology changes under different ionic conditions. The ^19^F-labeled nucleoside, however, gave sharp and position-specific signals for each structure. For example, upon increasing KCl concentration, the peak at −60.55 ppm, assigned to the parallel G4, increased and the integration of these peaks allowed the quantification of topology populations.

About the second novelty, the authors were able to demonstrate the utility of the probe in detecting the formation of G4 structures in cell-like environments using frog egg lysates. Unlike ^1^H NMR, which suffered from line broadening in complex samples, the ^19^F signal remained distinct, allowing for the structural characterization of G4s in situ. This demonstrated that the probe could be used to study G4 dynamics in cellular conditions as well.

**Figure 22:**
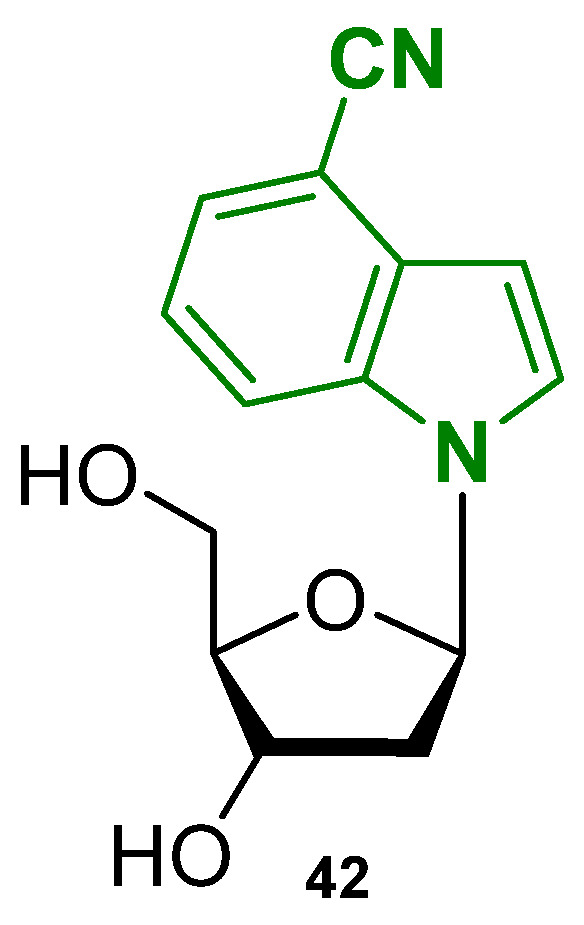
Structure of the nucleoside-based fluorescent small molecule 4CNI-NS (**42**).

A recent study from 2025 also explored similar strategies, introducing 4-cyanoindole-2′-deoxyribonucleoside (**4CNI-NS**) as a fluorescent nucleoside probe (Figure 22, compound **42**) [114]. In this work, **4CNI-NS** was incorporated into the human telomeric repeat by replacing specific thymine residues, allowing researchers to monitor G-quadruplex formation and conformational changes through changes in fluorescence intensity and lifetime, without significantly perturbing the native DNA structure, confirming once again the versatility of these techniques.

### 2.8. Other Small Molecules as Fluorescent Probes

**Figure 23:**
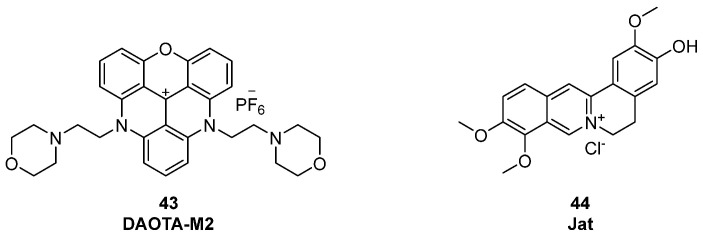
DAOTMA-M2 (**43**) and Jat (**44**), as new fluorescent probes used for G4 detection.

The group of Vilar et al. developed the triangulenium derivative turn-on probe **DAOTA-M2** (Figure 23, compound **43**) for which cell permeability, nuclear localization, and low toxicity were reported, making it suitable for live-cell imaging. After its first appearance in 2015 [115], extended studies were published in 2021 by the same group [116], who reported that by using a Fluorescence Lifetime Indicator Displacement Assay (FLIDA) with known G4 ligands (**PDS** or metal salphens), the displacement of **DAOTA-M2** was shown. Control experiments *in cellulo* using DAPI, a duplex binder that does not interact with G4, showed the specificity of the probe to quadruplex since the presence of DAPI in the cellular environment did not affect **DAOTA-M2**’s fluorescence lifetime. Furthermore, in the presence of cisplatin, a DNA-damaging agent, the observed fluorescence was also not affected, indicating that the modality of action of **DAOTA-M2** is not linked to general DNA damage but is rather specific for G4 structures.

Additionally, in a work by Zhang et al. from 2025, a label-free aggregation-induced emission (AIE)-based probe was described [117]; **jatrorrhizine chloride** (**Jat**) strongly lights up in the presence of antiparallel G4 DNA with about 20-fold fluorescence enhancement over other DNA types including duplex (Figure 23, compound **44**). Its non-disruptive binding was demonstrated by CD studies that did not show any change in the topology of the G4-forming sequences used. Furthermore, researchers created a fluorescence-based test system using Jat to detect DNA polymerase activity. In this context, adding a DNA polymerase to a hairpin DNA structure with hidden G4, the strand is extended by the enzyme releasing the quadruplex. After its release, Jat can bind to it and fluorescence is observed, thus indicating active polymerase activity.

**Figure 24:**
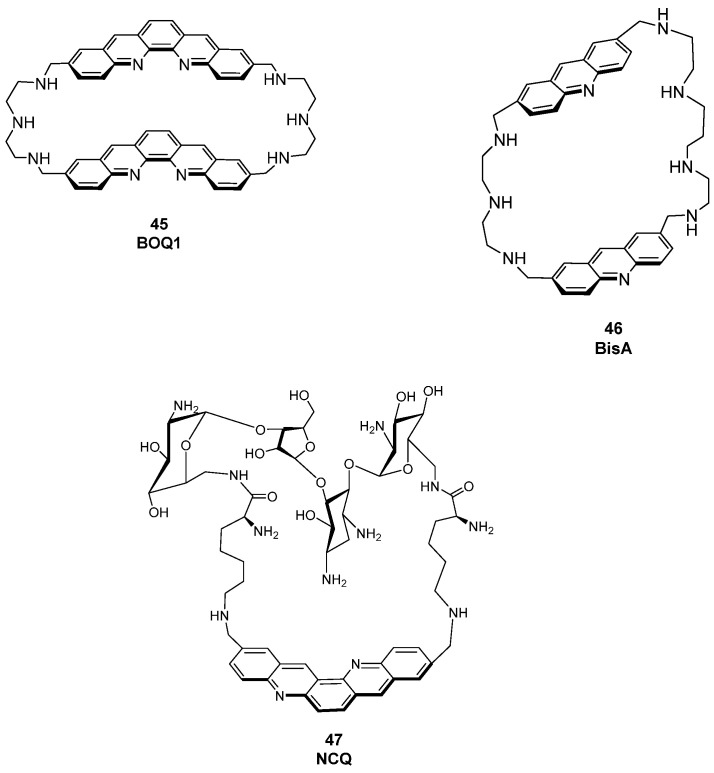
Structures of some known macrocycle derivatives used as fluorescent probes for G4 detection. CBIs (**45**, **46**) and NCQs (**47**) are reported.

More recently, attention has turned to cyclophane-like polyammonium macrocycles, a comparatively less explored class of G4 ligands that can be divided into two main families [118]: cyclobisintercalators (CBIs) and neomycin-capped aromatic platforms (NCQs). CBIs are composed of two large aromatic units (typically acridine or quinacridine) tethered by polyamine chains, a scaffold that confers high solubility at physiological pH and remarkable selectivity for G4 structures. Their semi-closed conformation prevents efficient duplex intercalation, favoring quadruplex recognition. Within this class, **BOQ1** and **BisA** have shown promising stabilization of human telomeric G4s, with ΔT_m_ values of approximately +28 °C and +15 °C, respectively (Figure 24, compounds **45**, **46**) [118].

In the NCQ family, a quinacridine platform capped with neomycin has been reported. This ligand displays poor duplex affinity and a clear preference for intramolecular (looped) G4s over tetramolecular ones, highlighting the potential of multitopic macrocyclic designs to achieve intra-G4 selectivity (Figure 24, compound **47**) [119,120].

## 3. Metal Complexes

In the context of the diverse conjugated systems to probe G4 in biological environments, metal-based complexes have emerged as powerful molecular tools for selective imaging and structural recognition. Thanks to their unique properties such as tunable luminescence, long lifetimes, and high photostability [121], metal complexes are preferred to traditional small organic molecules as DNA fluorescence probes and have been widely explored for G4 visualization. Generally speaking, the use of metal complexes on B-DNA has been previously explored [122] but researchers have later moved towards their application on non-canonical structures, through the insertion of metals bearing different electronic and coordination features, influencing the spatial disposition of the final ligand and its interaction with the genome. These complexes often display a binding selectivity for G4 over duplex DNA and can show different photophysical properties useful for diagnostic purposes. In fact, the insertion of a metal atom into a ligand scaffold leads to a photoactive system capable of emitting light upon binding to the biomolecular target, exhibiting “turn-on” behavior. Regarding transition metal complexes, they display phosphorescence behavior, and the observed phenomena are commonly of metal-to-ligand charge transfer (MLCT) character, involving an electron promotion from the metal-centered d orbital to a ligand π* orbital resulting in a long-lived excited state. When the metal complexes can be engineered to respond with “turn-on” emission upon binding, they can allow real-time monitoring of G4s [123].

### 3.1. Ruthenium Complexes

**Figure 25:**
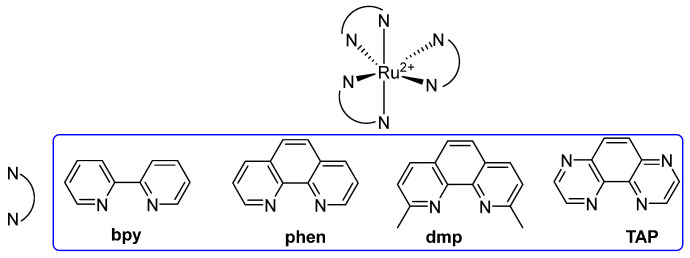
General structure of Ru(II) polypyridyl complexes employed as G4 probes. The prototype [Ru(bpy)_3_]^2+^ bears three bpy ligands, while different analogs were generated by introducing diverse substituents such as *phen*, *dmp*, and *TAP*.

A first class of metal-containing molecular tools for G4 detection is the one of the Ru(II) complexes, bearing this metal ion linked to “ancillary” ligands with different structural features. Throughout the years, different studies have been conducted to obtain new analogs with different features, with the aim of introducing useful and selective Ru(II) complexes to be used as probes. The first reported example in this class is represented by the **[Ru(bpy)_3_]^2+^**-containing 2,2′-bipyridine (*bpy*) group [65,124]. Diversely substituted ligands were later introduced, such as *phen* (1,10-phenanthroline), *dmp* (2,9-dimethyl-1,10-phenanthroline), and *TAP* (1,4,5,8-tetraazaphenanthrene), all belonging to the class of polypyridyl complexes (Figure 25).

**Figure 26:**
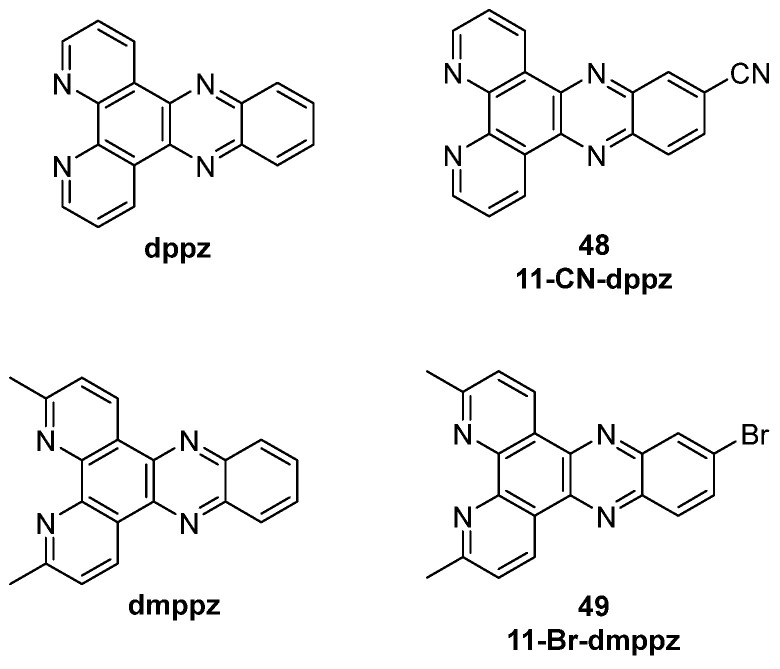
Structures of *dppz* and *dmppz* ligands with respective functionalization bearing a -CN group (**48**) and Br (**49**).

More specifically, **[Ru(bpy)_2_(dppz)]^2+^** and **[Ru(phen)_2_(dppz)]^2+^** (*dppz* = dipyrido[3,2-a:2′,3′-c]phenazine) were also reported and their behavior in water and upon DNA interaction was studied. In water, no emissions were observed, owing to the formation of H-bonds involving the nitrogen atoms of the ligands that are responsible for the quenching and stopping of luminescence, while in the presence of DNA the absence of H-bonds formation can lead to an effective emission, making them turn-on probes [65,125]. Studies to gain information on the interaction modalities between the complexes and different G4s in the solution were performed, showing changes in the behavior for different conformations of non-B-DNA sequences, and indicating that interactions can either happen at the loop regions, as for **Λ-[Ru(TAP)_2_(dppz)]^2+^**, or directly to the G tetrads, as observed for **Λ-[Ru(TAP)_2_(11-CN-dppz)]^2^**^+^, with the latter being the product of a cyano group insertion in the ligand structure. On the other hand, **∆-[Ru(phen)_2_(11-CN-dppz)]^2+^** was seen to bind to terminal T-T pairs instead (Figure 26, compound **48**). Structural modifications to gain SAR outcomes were performed. One of the studied products, **[Ru(bpy)_2_(dmdppz)]^2+^** bears a modified polypyridyl system, the *dmdppz* (3,6-dimethyldipyridophenazine). On this complex, the effects of light-induced ligand ejection were observed, an event in which upon light excitation, the ligand component of the complex is ejected, leaving the metal center behind and making it available for interaction with the DNA. This modality of action can be useful for photodynamic therapy and light-activated DNA targeting. Among other chemical modifications, brominations were useful to improve G4 selectivity, as observed with **[Ru(bpy)_2_(11-Br-dmdppz)]^2+^** (Figure 26, compound **49**) [126].

**Figure 27:**
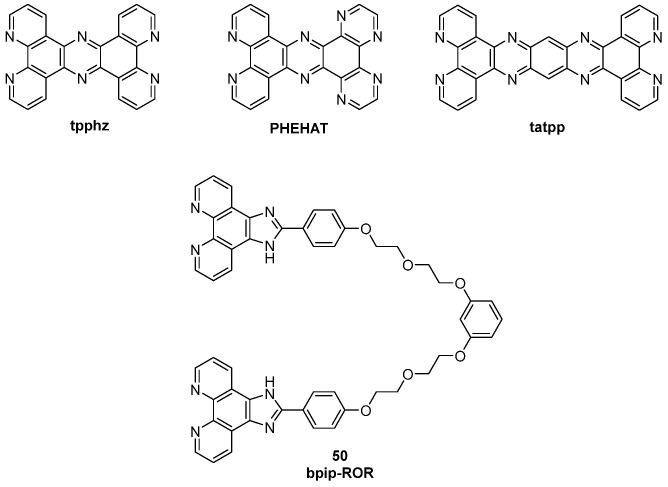
Structures of *tpphz*, *PHEHAT*, and *tatpp* ligands and bpip-ROR (**50**).

Apart from *dppz* ligands, extended moieties such as *dppzi* (dppz-imidazole) were reported but contrarily to the previous ones, they showed luminescence in water and no switch-on effect with DNA was observed, so they did not find any application as probes. Subsequently, researchers focused on the use of dinuclear Ru(II) to produce new tools. Thomas and co-workers characterized **[Ru(bpy)_2_}_2_(tpphz)]^4+^** and **[Ru(phen)_2_}_2_(tpphz)]^4+^** complexes, containing the *tpphz* (tetrapyridophenazine) moiety. For these compounds, an emission 2.5 times higher upon interaction with G4 rather than the duplex was observed and low cytotoxicity was found with IC_50_ = 138 μM on MCF-7 cells after 24 h. The two complexes also showed different behavior, making them useful for different purposes: **[{Ru(bpy)_2_}_2_(tpphz)]^4+^** only enters fixed cells while **[{Ru(phen)_2_}_2_(tpphz)]^4+^** can enter live cells, making the first one a death indicator, while the second one proved to be an innovative metal-complex probe thanks to its ability to localize inside the nucleus of the cell. **[{Ru(phen)_2_}_2_(PHEHAT)]^4+^** is a dinuclear complex, with a binding preference for antiparallel G4s although it lacks significant G4-over-duplex selectivity. On the other hand, complex **[{Ru(phen)_2_}_2_(tatpp)]^4+^** showed a very high affinity (K = 4.2 × 107 M^−1^) and selectivity, while among dinuclear complexes bearing the imidazo–phenanthroline group, **[{Ru(bpy)_2_}_2_(bpip-ROR)]^4+^** was the only one showing switch-on properties (Figure 27, compound **50**).

**Figure 28:**
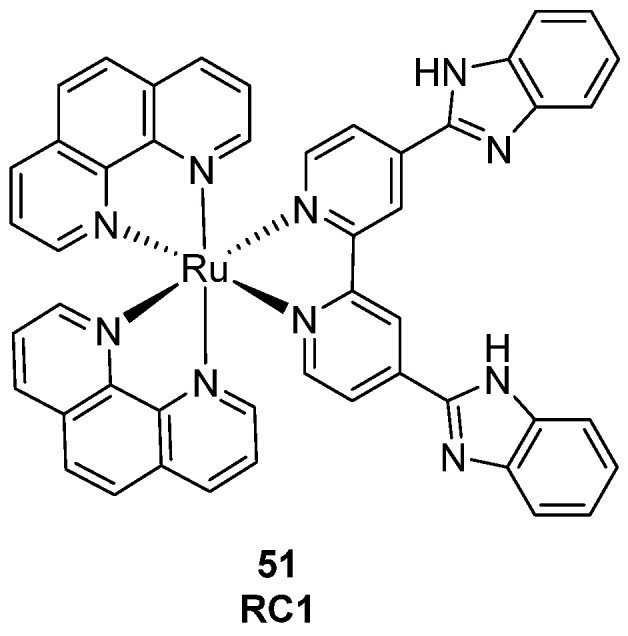
Structure of complex **RC1**.

In a very recent work from 2025, another Ru(II) complex was described, obtained through new structural modifications. Complex **RC1** (Figure 28, compound **51**) presents in its structure a two-armed benzimidazole ligand (*bimbpy*) that was first synthesized and then inserted into the complex to gain more selectivity on target G4 during intercalation. With this probe, a luminescence enhancement upon binding was detected, specifically with the HTDNA G4-forming sequence (6.1-fold increment). Its selectivity is thought to be mainly related to the rigidification of the two side arms after interaction with G4 DNA [127].

### 3.2. Platinum Complexes

Following the introduction of ruthenium in the structure of light-responsive G4 probes, platinum-based systems have emerged as a complementary class of metal complexes with different structures and photophysical properties. These reported compounds, particularly those with square-planar geometry, are well known for their ability to engage in strong π-π stacking and groove-binding interactions with nucleic acid structures, leading to genome modifications that are crucial for their mechanism of action. The positive findings of cisplatin as an anticancer agent paved the way to new platinum derivatives whose structures and modalities of interaction with DNA have been widely studied [122].

Apart from the use of these metal-containing compounds on canonical B-DNA, a strong focus was put on their affinity towards non-canonical structures and on their use not only as antiproliferative agents but as theranostic ligands as well. More specifically, several cyclometallated Pt(II) complexes have emerged as powerful tools and probes on G4s, especially thanks to their long phosphorescence lifetimes, topology-dependent emission properties and their application in phosphorescence lifetime imaging microscopy (PLIM).

**Figure 29:**
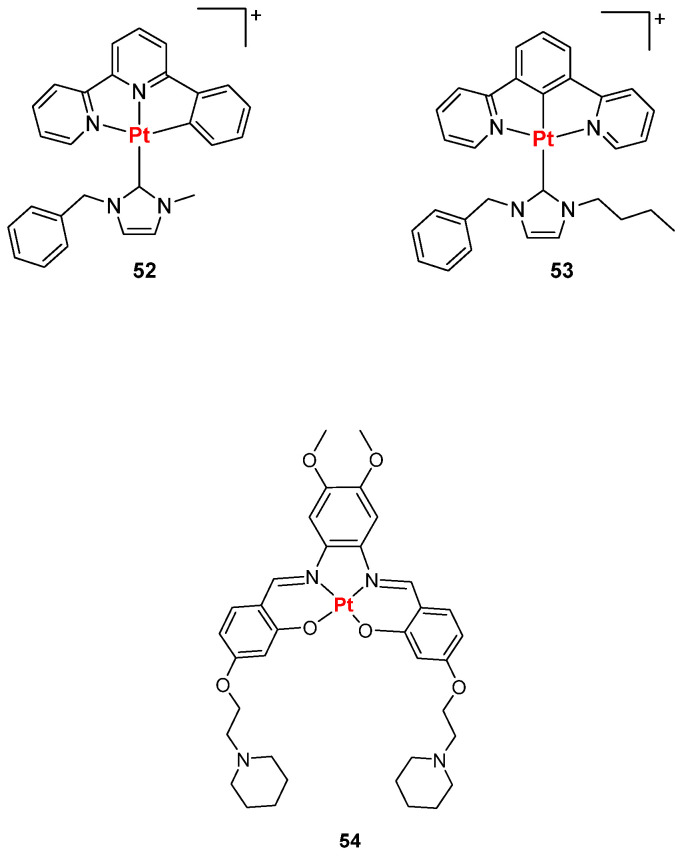
Structures of Pt(II) complexes bearing C^N^N, N^C^N (**52**, **53**) and O^N^N^O (**54**) pincher ligands.

Among the groups that have been involved in this research throughout the years, the one of Che developed complexes with C^N^N or N^C^N pincer ligands, bearing high selectivity and luminescence enhancement upon G4 binding (Figure 29, compounds **52**, **53**) [128]. On the other hand, complexes bearing different ancillary ligands to the O^N^N^O type, were obtained, with complex **54** being the best one in terms of interaction with *c-MYC* G4 DNA (Figure 29, compound **54**) [128].

**Scheme 1:**
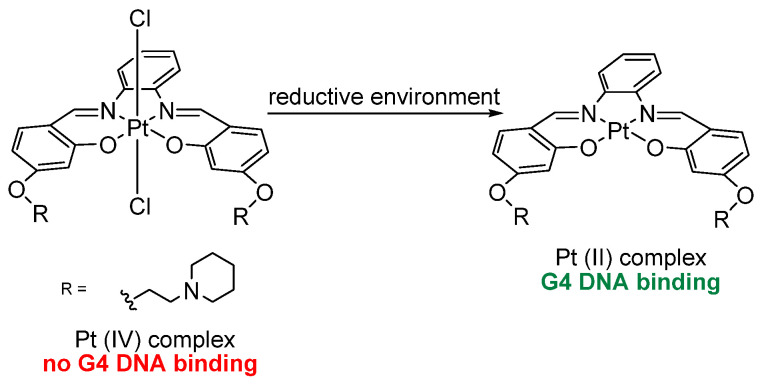
Redox-regulated Pt-based G4 binder. The octahedral Pt(IV)–salphen complex is reduced under hypoxic conditions to its Pt(II) form, thereby converting from a non-binding, non-emissive form into a G4-binding luminescent species.

Later on, Vilar et al. reported a redox-regulated G4 binder, based on an octahedral Pt(IV)–salphen complex that in reducing conditions, similar to the ones in hypoxic neoplastic tissues, is converted to Pt(II), going from a non-binding form, Pt(IV), to a binding one, Pt(II), and becomes emitting upon interaction (Scheme 1). The interconversion of the two forms resulted to be useful in cancer theranostics [129].

**Figure 30:**
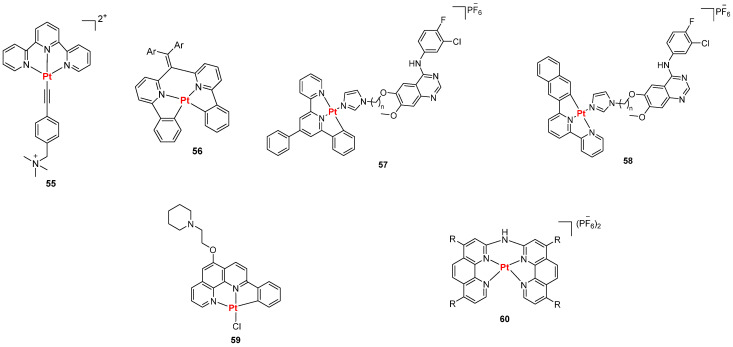
Structures of representative Pt(II) complexes reported as luminescent probes for G4.

Pt(II)-terpyridine complexes were also reported as luminescent probes by Yam et al., among which compound **55** (Figure 30) was patented in 2008, while Pigge et al. described Pt(II) complexes bearing tetraarylethylene-based ligands (Figure 30, general structure **56**), whose binding studies supported a minor-groove metalloinsertion mechanism [130]. A series of C^N^N cyclometallated platinum (II) complexes modified with anilinoquinazolines were described [131]. The conjugation of anilinoquinazoline derivatives related to the known epidermal growth-factor (EGFR) inhibitor gefitinib to cyclometallated platinum (II) complexes (Figure 30, compounds **57**, **58**), resulted in final dual-targeting agents that can both inhibit EGFRs, maintaining potent nanomolar IC_50_ values, and bind G4 DNA via the platinum complex with enhanced luminescence and affinity. Vilar et al. reported some phenanthroline-substituted Pt(II) complexes like compound **59** that gave positive outcomes in terms of selectivity towards G4 as its emission enhancement was higher in the presence of *c-MYC* G4 rather than duplex DNA. Other phenanthroline-based complexes were reported by He et al. [132], who described compound **60** and its positive probe abilities, especially towards G4 RNA. This was confirmed by BG4 immunofluorescence studies, where co-treatment with the G4-specific antibody BG4 revealed fluorescent foci overlapping with those of the complex in the cytoplasm, indicating its specific targeting of RNA G4s.

### 3.3. Iridium Complexes

**Figure 31:**
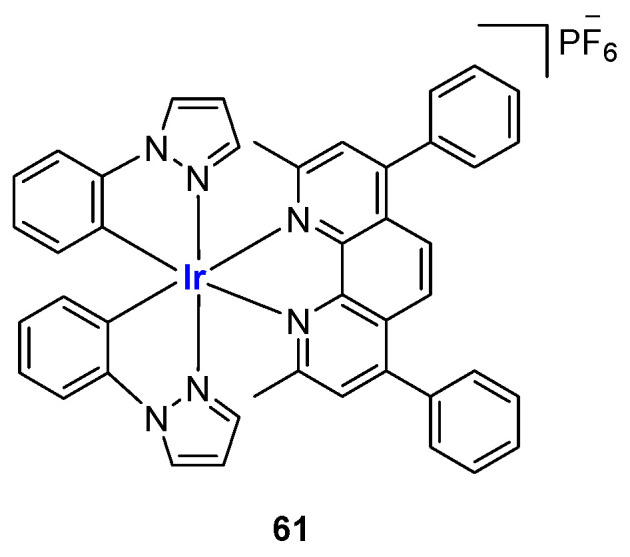
Structure of Ir(III) complex **61**.

Iridium (III) complexes have emerged as powerful tools in the G4 field thanks to their positive features such as long-lived phosphorescence lifetime, resistance to self-quenching, and consistent photoluminescence [133]. In addition, their easier synthetic development and higher air stability in comparison with platinum and ruthenium compounds have made them another important class of metal-containing complexes as probes on G4, with octahedral Iridium (III) complexes being the most widely used [134]. In 2013, complex **61** (Figure 31) was described as one of the first complexes capable of interacting with G4 structures, although its use was strictly related to the detection of the proof-reading activity of polymerases [135].

**Figure 32:**
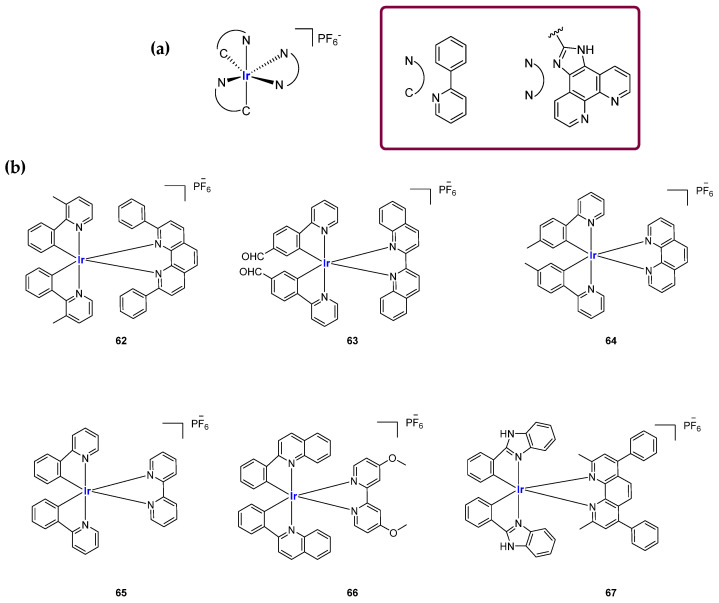
Structures of representative Ir(III) complexes reported as luminescent probes for G4. (**a**) G4 probes with general formula [Ir(ppy)_2_(N^N)]^+^. (**b**) Set of Ir(III) complexes studied for G4-based detection tools for adenosine. Compound **62** showed the best selectivity for the target.

In 2015, a set of different complexes was described. The general formula **[Ir(ppy)_2_(N^N)]^+^** indicates the presence of *ppy* (2-phenylpyridinato) and phenylimidazole phenanthroline as ancillary ligands (Figure 32a). For these analogs, studies reported a 50 times higher luminescence in the presence of G4 sequences, with micromolar affinity on telomeric G4 structures [136]. In 2016, another set of six complexes (Figure 32b) was described with **[Ir(mppy)_2_(2,9-diphen)]^+^** being the most selective G4 probe, that in this work was used for the detection of adenosine, an important purine nucleoside involved in diverse physiological functions across various systems including muscle contraction, blood flow regulation, and neuroprotection. In the absence of adenosine, an aptamer hybridizes with a complementary DNA strand to form a duplex, which interacts weakly with the complex. Upon binding to adenosine, the aptamer undergoes a conformational change, releasing the complementary strand, which then folds into a G4 structure that is strongly recognized by the probe, resulting in a luminescence switch-on signal [137].

**Figure 33:**
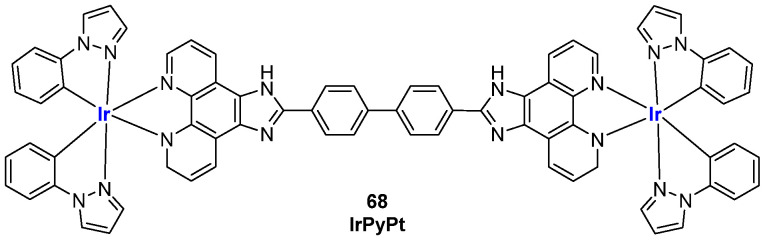
Structure of complex IrPyPt (**68**).

The ability to interact with G4 structures by these iridium complexes was also studied by Li et al., who described the complex **IrPyPt** (Figure 33, compound **68**), used for the detection of microRNA containing G4, confirming once again the use of Iridium (III)-based probes for the detection of bioanalytes [138].

**Figure 34:**
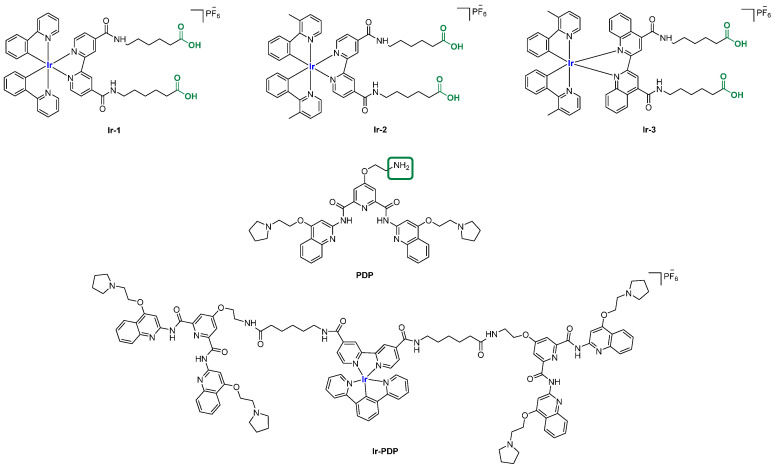
Structures of three Ir (II) complexes (**Ir-1**, **Ir-2**, **Ir-3**) used for conjugation with a known G4 binder (**PDP**). The carboxylic and amine groups of the two counterparts available for coupling reactions are reported in green. One of the resulting products studied as a G4 probe is reported as **Ir-PDP**.

Later on, in 2023, conjugates with known G4 ligands, the pyridostatin analog PDP, and Iridium (III) were obtained, leading to Ir-PDP. The described workflow is based on a functionalization of the Ir(III) scaffolds with carboxylic groups, leading to a small group of three analogs, Ir-1, Ir-2, and Ir-3, later involved in coupling reactions with PDP bearing a primary amine group. The final compound Ir-PDP (Figure 34) was described as a phosphorescence turn-on probe with a cytoplasm distribution inside the cell and can be considered an example of metal complex conjugated with a G4 binder [139].

### 3.4. Lanthanide Complexes

**Figure 35:**
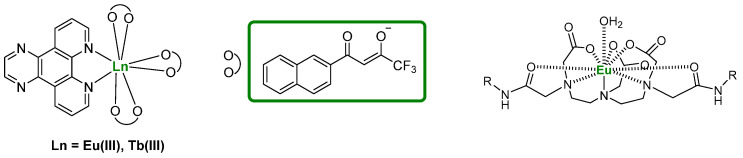
General structures of lanthanide metal complexes used for G4 detection.

Although less extensively studied than transition metal compounds, luminescent lanthanide complexes have been employed as DNA optical probes thanks to their positive features such as large Stokes shifts and long excited-state lifetimes which enable time-gated acquisition techniques [140]. To properly function, the structures of these derivatives are usually enriched by “antenna” chromophores that absorb energy from radiation and transfer it to the general Ln(III) ion [141]. The interaction of lanthanide ions with G4 DNA has been studied for over two decades, involving metal complexes containing Tb(III) and Eu(III) (Figure 35) [123,142]. Chatterji et al., for instance, reported that the luminescence of Tb^3+^ can be effectively enhanced by the G4 DNA of the tetrahymena telomeric sequence [143]. In more recent works, lanthanide complexes have been employed as structure-specific G4 probes. For example, taking advantage of the hydrolytic activity of Ce^4+^, Komiyama and co-workers designed a Ce^4+^-EDTP (ethylenediaminetetramethylenephosphonic acid) complex covalently attached to the 5′-end of a DNA probe containing three G-tracts, in which a syn-conformation dG was replaced with 8-bromoguanosine. This engineered DNA-EDTP-Ce^4+^ probe assembles into a 3 + 1 intermolecular G4 with human telomeric DNA and once the G-quadruplex is formed, the Ce^4+^ complex is positioned against a specific phosphodiester site, enabling sequence-specific cleavage of the telomeric DNA through hydrolysis. By selectively cleaving DNA only where a G4 is formed, researchers can identify the exact sequences that adopt G4 structures in a complex DNA sample and this is useful for understanding telomere biology or gene regulation where G4s play a role [144]. Furthermore, DOTASQ-Tb(III) complexes obtaining the active form of the guanine-containing analog are also known. In this case, the coordination with the ion shifts the equilibrium towards the active closed conformation of DOTASQ, ensuring its binding activity (see Section 2.5) [145].

## 4. Conjugated Molecular Tools

In recent years the growing interest in G4 has driven the development of increasingly complex and engineered systems, requiring more sophisticated and versatile molecular tools aimed at gaining new genomic information. Among the design and synthetic strategies employed, conjugation procedures between different components, both of chemical and biological interest, were described and were crucial to lead to the development of hybrid molecular tools whose modality of action is based on the synergic combination of the single parts. Agents belonging to this class can enable not only the stabilization or disruption of G4 structures to regulate biological outcomes but also their real-time detection and localization in cells and tissues. Such conjugates are proving to be useful tools in elucidating G4 dynamics in physiological and pathological contexts, especially in oncology fields where, as earlier described, G4s are enriched in promoter regions of oncogenes and in telomeres.

### 4.1. Strategies for Ligand Conjugation and Functionalization

By employing traditional chemical modifications and specific functional groups, researchers have applied conjugation strategies focusing on the use of modulable linkers and simpler organic chemistry approaches involving the use of well-known and already available G4-interacting moieties as starting points. These classical conjugation methods enable precise tuning of the behavior of the obtained compound.

**Figure 36:**
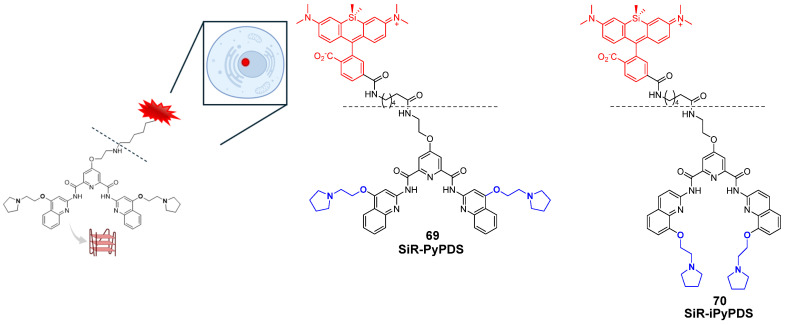
Structures of two products of conjugation studied as G4 probes. The silicon–rhodamine (**SiR**) fluorescent moiety is reported in red. SiR-PyPDS (**69**) resulted from the conjugation with PyPDS to enable both selective G4 binding and fluorescence-based detection at low nanomolar concentrations. SiR-iPyPDS (**70**) was obtained through conjugation with iPyPDS and it was used as a negative control.

The first example can be found in a work by Di Antonio et al. [146], through the design of **Sir-PyPDS**, a product of conjugation between the fluorophore silicon–rhodamine (SiR) and the G4 ligand **PyPDS**. The aim was to combine the binding activity of the ligand with the fluorescence properties of the SiR moiety, which was useful for detection. The advantage of this method is linked to the real-time visualization of G4 structures at low nanomolar probe concentrations, minimizing the perturbation on the dynamics of the non-canonical structures. Different structural modifications including studies on the length of the linkers were made to optimize the activity of the analogs with compound **Sir-PyPDS** (Figure 36, compound **69**) being the most promising with K_d_ values of 0.63 ± 0.08 μM, 1.0 ± 0.1 μM, and 2.0 ± 0.8 μM, on *c-MYC*, *c-KIT1*, and *h-TELO* G4s, respectively, and no binding on non-G4 DNA, showing selectivity on the target. Studies using a negative control were performed through the design of **Sir-iPyPDS** (Figure 36, compound **70**) which does not show the ideal spatial arrangement to interact with G4 structures.

The evaluations were based on the number of observable binding events that visually correspond to fluorescent dots obtained when a single probe can bind to the G4 structure and keep the interaction for enough time to be detected. Single-molecule imaging in vitro showed that **Sir-PyPDS** binds to immobilized *MYC* G4 at very low concentrations (250 pM) while the isomer **Sir-iPyPDS** produced a 10-fold reduction in binding events. The specificity was further supported by a strong binding reduction upon Phen-DC3 competition and when a mutated *MYC* sequence incapable of G4 formation was used, confirming the suitability of the complex for G4 detection. For in vivo studies, the two compounds were tested on U2OS cells and no toxicity was observed at the nanomolar concentrations needed for imaging. At tolerable concentration of 20 nM of **Sir-PyPDS** “under-labeling” G4 was observed, meaning that no crowding in the detection was seen with well-separated individual dots. Around 79 specific binding events per cell nucleus were seen with fluorescent spots indicating probes bound to the G4. Control compound **Sir-iPyPDS** only showed ~2 binding events. Furthermore, differences in binding were not due to variations in probe uptake, as **SiR-PyPDS** and **SiR-iPyPDS** showed similar cellular entry at 10 µM. As with during the in vitro experiments, and also in this case, competitions assays using PDS and Phen-DC3 decreased the signal confirming the G4 binding. Interesting outcomes on the use of the conjugated probe were related to the study of G4 dynamics using dimethyl sulfate (DMS). This compound can methylate the N7 position of guanine bases in unfolded G4s only, because in folded conditions, those positions are not accessible for the chemical modification caused by DMS. During in vitro studies, a strong reduction in binding events by **Sir-PyPDS** before and after DMS treatment was registered, showing that DMS blocks the formation of G4 preventing the binding of the probe. In living cells, the drop in G4 detection was time dependent. This can be explained with the fact that in normal cellular environments, G4s have dynamic behavior and can unfold, temporarily exposing guanine bases to DMS. To further assess this, more studies were performed on the cell cycle; during the S phase, a high number of binding events were observed, demonstrating that G4s appear during active DNA events like replication and transcription and not when the cell is resting, since in the G0/G1 phase a lower number of binding events was registered (~3). U2OS were also treated with replication and transcription inhibitors (aphidicolin and 5,6-dichloro-1-β-d-ribofuranosylbenzimidazole, DRB) to recreate a G0-like inactive state, and the reduced number of binding events was confirmed.

**Figure 37:**
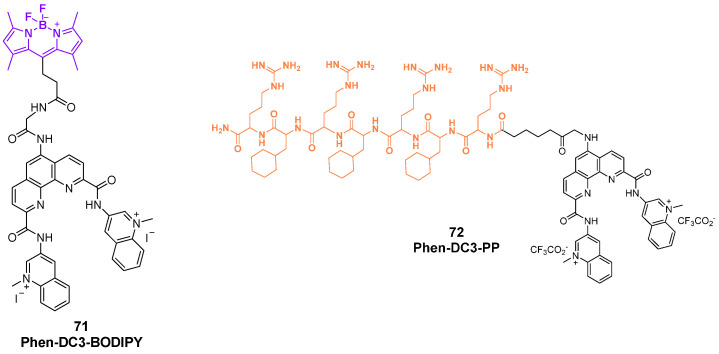
Structures of the products of conjugation between Phen-DC3 and BODIPY (**71**) and the cell-penetrating peptide (**72**).

A work proposed by Prasad et al. can also be considered as key to understanding the versatility of conjugation approaches that involve known G4 stabilizers to convert them from simple binding agents into useful tools [147]. Phen-DC3 was picked for a series of chemical modifications: once addressed the available positions for structural changes, the ligand was functionalized with different conjugation strategies with the first modification consisting of the hybridization to **BODIPY**, a known fluorescent dye with the aim of converting Phen-DC3 into the **Phen-DC3-BODIPY** chimera, useful for G4 detection (Figure 37, compound **71**). After the synthetic optimization, the first experiments were conducted to confirm that the conjugation did not affect either G4 stabilization—as checked through Taq-Pol assays—or the cellular properties of the starting ligand Phen-DC3, since the final product retained similar cell viability effects as the unmodified ligand. Linking Phen-DC3 with BODIPY helped to gain insights through live-cell imaging of the localization inside the cellular environment, with the detection of its presence in the mitochondria area and not inside the nucleus, thus explaining the limited effects on nuclear DNA. Poor nuclear entry and limited overall uptake pushed for conjugation with new useful chemical moieties. Phen-DC3 was indeed conjugated to a cell-penetrating peptide (CPP), affording **Phen-DC3-PP**, to observe any enhancement in the permeation abilities of the final hybridized tool (Figure 37, compound **72**). The synthetic efforts and molecular characterizations were described, and after assessing no changes in the stabilization effect of the Phen-DC3 component, the conjugate **Phen-DC3-PP** was proved to have stronger effects on cell viability for the synergic activity of the two parts, since the CPP alone did not have any effect on the cells. The presence of the peptide helped penetrate inside the nucleus, thus affecting nuclear G4 DNA. This was confirmed by monitoring the phosphorylation of H2A.X, a marker of nuclear DNA double-strand breaks, through which **Phen-DC3-PP** caused DNA damage in HeLa cells at 12.5 μM, while unmodified Phen-DC3 only had a moderate effect even at higher doses (100 μM).

**Figure 38:**
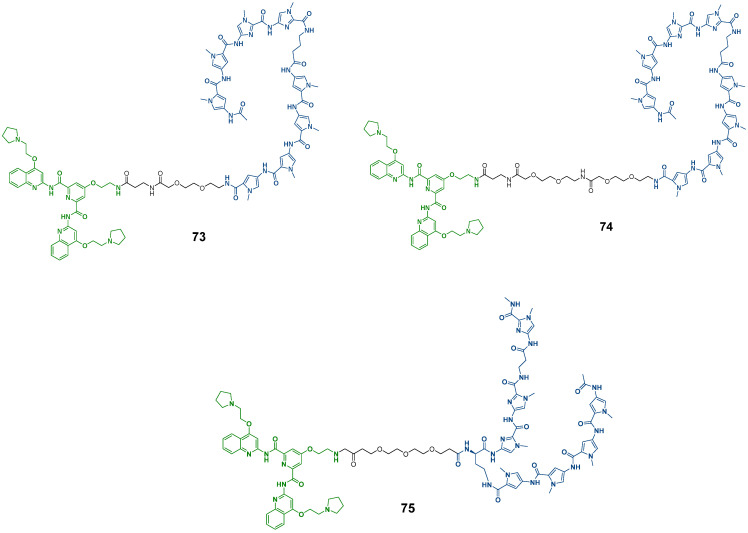
Structures of probes used for G4 detection obtained through conjugation between **PyPDS** (in green) and pyrrole–imidazole polyamide (**PIP**) moieties (in blue).

The concept of conjugated molecular tools as probes should extend beyond the traditional meaning related to G-quadruplex detection. These systems should be indeed considered not just as passive reporters but also as active agents capable of modulating G4 selectivity. Through thoughtful design—whether via ligand architecture, conjugation to oligonucleotides, or integration with responsive units—such molecular probes can be tailored to enhance their discriminatory power among different G4 topologies, or even between G4 and non-G4 structures. This dual functionality, encompassing both recognition and selective stabilization, can turn molecular tools into versatile instruments for fine-tuning G4-targeted strategies in both analytical and therapeutic contexts. As proof of this concept, in a work by Ooga et al. [148], the conjugation strategy was used to increase the selectivity of known G4 binders by linking the G4-binding moiety of interest to another structural component able to interact with a duplex region in proximity to the target G-rich one. The synergic action of these two parts was responsible for the incremented selectivity of the stabilization effect, since the duplex-interacting part directed the ligand to a specific G4 sequence. Three different conjugates (Figure 38, compounds **73**, **74**, and **75**) were built and characterized using the known G4 ligand **PyPDS** and two different **pyrrole–imidazole polyamide** (**PIP**) molecules, which act as duplex DNA-binding sequences. Through the use of different linkers, the two parts were conjugated, and the hybrids were next tested to observe their stabilization effect on both the *c-MYC* G4-forming sequence and the close duplex sequence that builds the NHEIII (Nuclease Hypersensitive Element), making a comparison with the single components alone.

**Figure 39:**
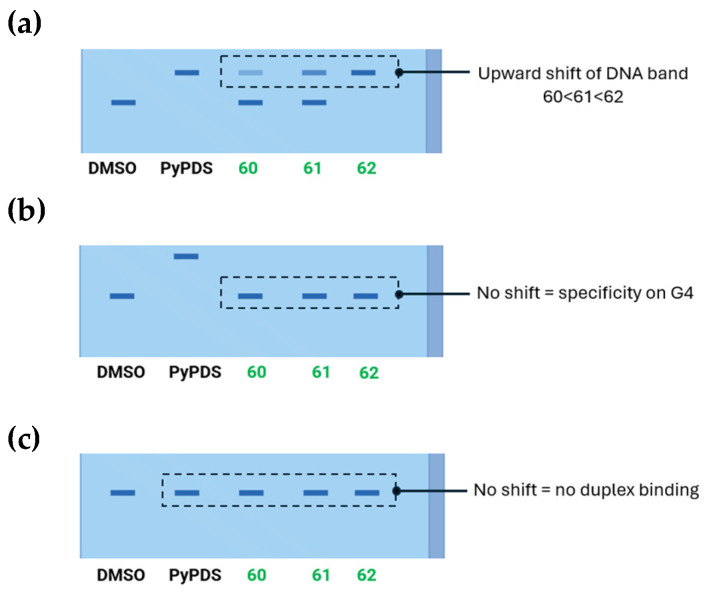
Schematic representation of native gel electrophoresis. A volume of 5 mol eq. of each conjugate compound was used with the target G4-forming sequence (**a**), non-target G4-forming sequence (**b**) and non-G4-forming sequence (**c**). The upward shift in the DNA band can be observed in the presence of the target sequence with the strongest intensity for conjugate **75**. The image was reproduced from ref. [134].

Through CD melting studies, conjugate **75** emerged as being the most stabilizing on G4s with ΔTm= +7.6 °C, outperforming **PyPDS** alone (ΔTm = +6.5 °C). The stabilization effect on the duplex was also reported with the same conjugate being the most effective. FRET assays confirmed these findings and computational models suggested that the PyPDS moiety in the conjugate binds at the G4–duplex interface, supporting dual recognition. More findings on the selectivity of **PyPDS-PIP** conjugates were obtained through an Electrophoretic Mobility Shift Assay (EMSA) using the target G4 sequence, a non-target G4 sequence and duplex DNA. In this experiment, the sequences were incubated with each conjugate and then ran on a native gel. The binding of the conjugates to DNA resulted in upward shifts in the DNA bands confirming interactions, and control experiments with non-target or non-G4 sequences showed no significant shift after incubation with the conjugates, demonstrating the high selectivity for the target (Figure 39).

Fluorescence displacement assays with ThT (a G4 probe) and EtBr (a duplex probe) also showed the selectivity for target G4s, taking into account the DC_50_ value, hence the concentration at which 50% of the fluorescence indicator is displaced, and the S value which is the ratio between DC_50_ for the non-target and for the target sequence. One of the conjugates had the highest S value in ThT assay (S = 2.3) against the lower value of **PyPDS** alone, showing a lack of selectivity (S = 1). In the EtBr assay, the conjugates showed a higher S value, confirming the selectivity on the duplex, compared to **PyPDS**. All the results confirmed that **PyPDS-PIP** conjugates efficiently and selectively bind the target G4 DNA flanked by duplex regions.

**Figure 40:**
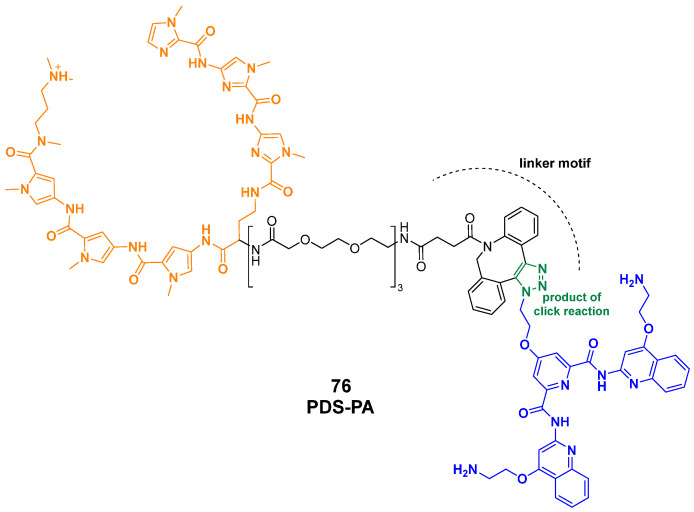
Structure of the PDS-PA hybrid conjugate (**76**) obtained through combination of G4-ligand PDS (in blue) and a sequence-specific binding polyamide PA (in orange). The molecule was obtained through the solid-phase synthesis of PA, linker incorporation, and a click-reaction using azido-modified PDS.

Similarly, in 2024 Karna et al. described new conjugates obtained by combining G4-ligand PDS and Polyamide (PA), a sequence-specific duplex binder, affording **PDS-PA** hybrids (Figure 40, compound **76**) [149]. As described with the previous workflow, the binding behavior of both single components and final conjugates were studied on the hTERT 5–12 promoter region, but the novelty of this work regarded the use of optical tweezers that showed how the two parts can influence the secondary structure of the picked sequence, with PDS leading to tandem G4 and PA promoting the formation of a hairpin–G4 hybrid by binding the duplex loop. **PDS-PA** final conjugates induced mixed folding patterns through the combination effect of the parent compounds. Other complementary studies like polymerase stop and luciferase gene assays showed that **PDS-PA conjugates** can cause stronger replication blockage and a more increased gene suppression regulated by hTERT than PA and PDS alone, confirming the synergistic binding.

### 4.2. Conjugation with Oligonucleotides and Aptamers

In addition to traditional strategies involving the functionalization of ligands with generic chemical groups, the selectivity of bio-conjugates can be significantly enhanced by conjugating the ligands to oligonucleotides or aptamers [150,151].

**Figure 41:**
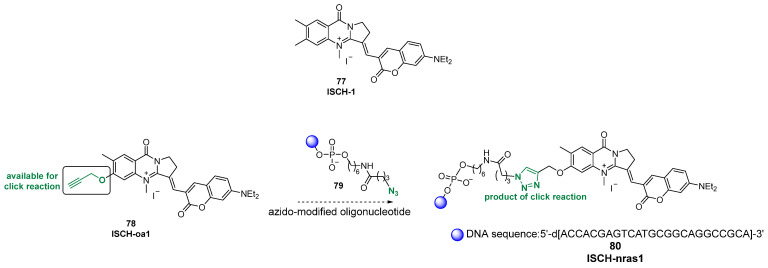
Structure of precursor ISCH-1 (**77**), its alkyne-functionalized derivative ISCH-oa1 (**78**), and the G-quadruplex-triggered fluorogenic hybridization (GTFH) probe ISCH-nras1 (**80**). The scheme illustrates the synthetic strategy based on the late-stage click conjugation of ISCH-oa1 with an azido-modified oligonucleotide to generate the final compound used for the selective recognition of G4s.

A work by Chen et al. reported the G4-triggered fluorogenic hybridization (GTFH) probe, named **ISCH-nras1** as a tool for the identification of G4 structures in RNA. This work was produced starting from a dual colorimetric and red-emitting fluorescent probe **ISCH-1** (Figure 41, compound **77**), previously reported by the same group that exhibited turn-on emissive properties upon G4 binding. **ISCH-nras1** was also tested in live and fixed cells showing strong nuclear fluorescence that was lost in the nucleoli, where ribosomal nucleic acids are produced. After DNase treatment, no fluorescence was observed, indicating that the interaction with **ISCH-1** involves G4 in the structures of rDNA since RNase treatment did not lead to emissions changes [152]. From **ISCH-1**, new studies were performed to obtain more selective probes to help discriminate among different G4 structures. **ISCH-1** was indeed first modified on accessible positions of the aromatic core through the insertion of an alkyne group (Figure 41, compound **78**) in order to favor a late functionalization with an azido-modified oligonucleotide sequence (Figure 41, compound **79**). The two components were hybridized through click chemistry, leading to a conjugated molecule composed of the G4 light-up fluorophore and a DNA strand complementary to the tail region adjacent to G4T25, in order to study the selectivity towards G4T25, a known G4-forming sequence located in the 5′-UTR of the NRAS mRNA. The activity of **ISCH-nras1** (Figure 41, compound **80**) in vitro with G4T25 confirmed the emission of fluorescence, while control RNAs with mutated or deleted G-rich sequences, produced minimal or no fluorescence. In cells transfected with the RNA of interest, fluorescent staining with **ISCH-nras1** showed specific cytoplasmic spots indicating the detection of intact G4 sequences, while the evaluation using mutated or deleted G-rich sequences produced minimal or no fluorescence. Treatments with RNase or the addition of complementary sequences to G4T25 that could prevent interaction with the probe abolished the signal-confirming specificity. Furthermore, competition assays using G4c, the complementary strand to the G-rich sequence, and IZCM-7 able to bind NRAS G4, showed a fluorescence decrease, confirming the activity of the probe. **ISCH-nras1** could also detect the G-quadruplex not only in the isolated G4T25 but in the full-length (254-nt) NRAS 5′-UTR RNA as well [153].

**Figure 42:**
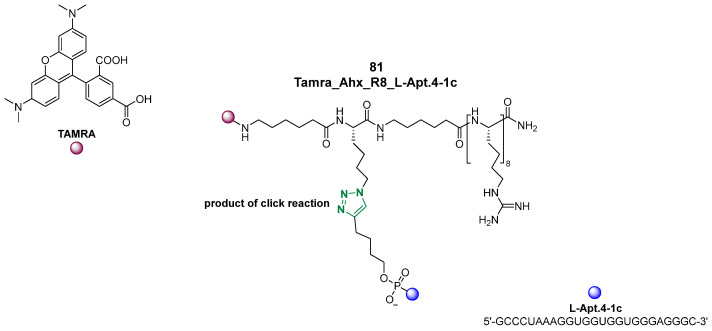
Structure of Tamra_Ahx_R8_L-Apt.4-1c (**81**) resulted from bioconjugation of the G4-binding aptamer L-Apt.4-1c (in blue) with a polyarginine cell-penetrating peptide (R8) via a 6-aminohexanoic acid (Ahx) linker and functionalized with TAMRA (in red).

In a work by Zhang et al. [154], another conjugate named **Tamra_Ahx_R8_L-Apt.4-1c** was reported as a product of the bioconjugation between a L-aptamer and cell-penetrating peptides (CPPs). Aptamers are single-stranded RNA or DNA oligonucleotides that can fold up and interact with different biological systems [155]. The first aptamers were described around 1990 and were obtained through the SELEX (Systematic Evolution of Ligands by Exponential Enrichment), a high-throughput in vitro strategy that starts from randomly built oligonucleotide libraries leading to biostable aptamers through three main steps, including the incubation of the library of nucleotides with the desired target, the separation of the target-bound aptamers from the unbound ones, and the final amplification step of the selected aptamers [156]. The widespread adoption of aptamers has been driven by their diverse positive features, positioning them as valid substitutes of antibodies; the two are often compared, as both work through selective and specific binding to target molecules. Aptamers can indeed recognize a series of biological targets, including G4, with high affinity and specificity. Furthermore, they show good thermal stability and, contrarily to antibodies, can be easily synthesized and functionalized through solid phase and automation procedures as well, with easy scale up, high efficiency, and low impact [157]. In the work by Zhang et al., the aptamer L-Apt.4-1c, known for its G4-binding activity but scarce cell permeability, was conjugated to CPPs, often used to deliver biomolecules into the cellular environment due to their involvement in endocytosis processes that help penetration. The final chimera **Tamra_Ahx_R8_L-Apt.4-1c** was described, bearing a fluorescent label for imaging (Tamra), a 6-aminohexanoic acid linker to enhance flexibility (Ahx), an 8-polyarginine CPP (R8), and L-Apt.4-1c (Figure 42, compound **81**). Once the chemistry was optimized and the final compound was fully characterized, studies were made to gain insight on the activity of the final probe. Different modifications were performed throughout the process changing CPPs, linker length, and TAMRA position but the named compound showed the strongest binding (K_d_ = 707.4 ± 64.6 nM). After assessing the binding activity of the complex through an EMSA, cellular uptake was also evaluated. When cells were pre-transfected with FAM-labeled *hTERC* rG4 WT, a fluorescent-tagged guanine-rich RNA sequence, the conjugate entered the cytosol and localized with the colocalized FAM signal target. In contrast, no signals were observed without transfection or with a mutant rG4, confirming target specificity. The regulation on gene expression was also assessed using an EGFP (Enhanced Green Fluorescent Protein) plasmid that was modified with *hTERC* rG4 WT or with a mutant sequence. After transfection and without the conjugate, the EGFP signal from the WT was weaker than the Mut case, meaning that the rG4 structure can reduce the expression of the detectable protein. On the other hand, in the presence of the conjugate, EGFP expression from the WT construct was about 73% lower while the Mut construct was not affected. This indicated that *hTERC* rG4 lowers protein production with a stronger effect in the presence of **Tamra_Ahx_R8_L-Apt.4-1c**. A dual luciferase assay was performed to observe any effects on the translation processes. Control experiments using Tamra_Ahx_R8 peptide alone or L-Apt.4-1c alone did not alter luciferase activity, indicating that both components are required for specific effects on the quadruplex. Interesting experiments were performed to asses whether **Tamra_Ahx_R8_L-Apt.4-1c** could regulate gene expression via the rG4 motif in the 3′-UTR of the amiloyd precursor protein (APP) mRNA, a gene linked to Alzheimer’s disease. *APP* rG4 WT or Mut was included in the 3′-UTR of the Renilla luciferase gene; the presence of the rG4 motif reduced reporter expression by 22% ca. and treatment with **Tamra_Ahx_R8_L-Apt.4-1c** further suppressed translation only in the WT rG4 construct with no effect in the case of the mutan construct.

**Figure 43:**
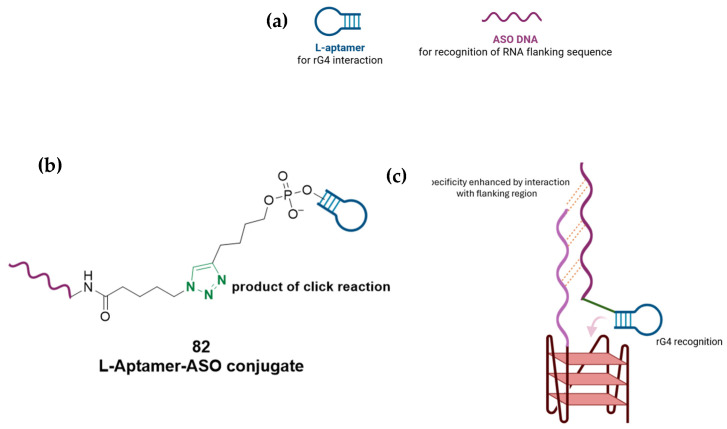
(**a**) Schematic representation of the L-aptamer and ASO DNA used for this conjugation strategy. (**b**) The final L-Aptamer–ASO conjugate (**82**) obtained through a click reaction. (**c**) Recognition of rG4 is possible through the L-aptamer moiety while the specificity is enhanced through the ASO sequence complementary to the flanking region of the target.

In a work from Zhao et al. published in 2024, the conjugation of the aptamer involved an antisense oligonucleotide (ASO) [158]. Specifically, an L-RNA aptamer was used for rG4 recognition and its selectivity was enhanced by conjugation with ASO DNA complementary to the target rG4-flanking region leading to the L-aptamer–ASO conjugate **L-Apt.4-1c-ASO15nt_(*APP*)_** that recognizes APP G4 (Figure 43, compound **82**). Specificity of the conjugate for APP G4 over other G4s like Bcl2, TRF2, MT3-MMP, and duplex was studied and **L-Apt.4-1c-ASO15nt_(*APP*)_** was considered to be the first L-aptamer–ASO conjugate for G4 imaging inside live cells. Interesting experiments were conducted to study a potential protein interaction disruption; the conjugate indeed blocked the binding to G4 of DHX36, an unwinding helicase, showing an additional mechanism of G4-mediated gene regulation.

**Figure 44:**
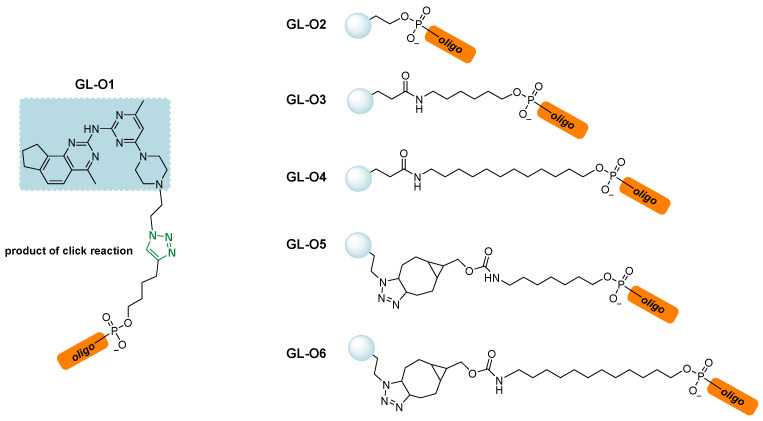
Conjugate **GL-O1** with G4 ligand moiety in light blue. Subsequent derivatives **GL-O2-O6** were obtained to study linkers effect on G4 stabilization.

Chorell and co-workers also developed GL-Os (G4 ligand-conjugated oligonucleotides) by combining a guide oligonucleotide, which directs the construct to a specific genomic location, with a G4-binding ligand that stabilizes the target structure. The first prototype, **GL-O1** (Figure 44), demonstrated the synergistic effect of these two components, providing both specificity and stabilization [159]. In a subsequent study, the strategy was refined by systematically varying the linker that connects the oligonucleotide and ligand [160]. Different synthetic approaches were explored—including amide coupling, copper-free click chemistry, and solid-phase oligonucleotide synthesis (SPOS)—to generate a versatile library of conjugates (Figure 44, **GL-O2-6**). Biophysical assays with the *c-MYC* Pu24T G4 showed that while all compounds preserved binding and stabilization, the length of the linker was more critical than its chemical nature. In particular, longer linkers, especially those obtained via SPAAC click chemistry, enhanced G4 stabilization even under thermal stress and when the guide sequence was positioned further from the G4. Short linkers, by contrast, lost efficiency as the distance increased. These experimental findings were reinforced by computational modeling, which revealed that all GL-Os could, in principle bind the G4, but shorter, more rigid linkers struggled to adopt favorable conformations for stable interactions. Longer linkers provided the necessary flexibility to maintain binding across different spatial arrangements, especially in complex genomic regions where multiple G4s or sequence variations exist. Together, these studies highlight both the potential of GL-Os as molecular probes and the central role of linker design in their optimization. Importantly, this platform is adaptable to fluorescent ligands, expanding its use toward live-cell G4 detection and functional studies, while also pointing to therapeutic applications at the DNA level.

## 5. Conclusions

G4s are specialized four-stranded DNA or RNA structures formed in guanine-rich regions of the genome and composed of stacked guanine tetrads stabilized by Hoogsteen hydrogen bonds and monovalent cations. They play crucial roles in regulating gene expression, maintaining telomere integrity, and controlling DNA replication and repair. Due to their involvement in such key biological processes, the development of molecular tools to study G4 structures has become increasingly important, since understanding G4 formation and dynamics can reveal insights into their biological functions and involvement in diseases such as cancer. Precise probes enable the visualization, detection, and manipulation of G4s in living cells, facilitating targeted therapeutic strategies. Additionally, molecular tools aid in elucidating the mechanisms by which G4s influence transcription and replication processes. Overall, these advances enhance the study of G4 structures and exploit their potential biomedical and therapeutic applications. In light of these considerations, this review provides an updated overview of the molecular tools developed for G4s, including fluorescent probes, metal complexes, and more recent conjugates.

Fluorescent probes, which comprise carbazoles, benzothiazoles, porphyrins, phthalocyanines, polyphenyls, G-clamp derivatives, and guanine-containing probes, proved to be essential tools for studying G4s due to their high sensitivity and specificity. These probes enable real-time visualization and detection of G4 structures within live cells and complex biological environments, aiding in understanding their formation, stability, and biological functions. By emitting fluorescence upon binding to G4s, they allow dynamic changes and interactions at the molecular level to be monitored. This insight is crucial for elucidating G4s’ roles in gene regulation, genome stability, and disease mechanisms, particularly in cancer. Overall, fluorescent probes have significantly advanced G4s’ investigation with precision and spatial resolution.

In parallel, complexes of metals such as ruthenium, platinum, iridium, and lanthanides have been developed as probes to visualize, stabilize, or induce G4 formations, aiding in structural characterization and biological studies. Although metal complexes exhibit valuable properties, their application in biological settings has been often hindered by several limitations, including potential cytotoxicity, solubility issues, and limited chemical flexibility for further functionalization. Additionally, the relatively large size and complex coordination geometry of many metal centers can impair cellular uptake and target accessibility.

To expand the range of accessible tools, researchers have also adopted conjugation strategies that utilize conventional chemical modifications and functional groups. These approaches emphasize the use of adaptable linkers and straightforward organic chemistry techniques, often relying on well-established and readily available G4-interacting components as initial building blocks. Such traditional conjugation methods allow for fine control over the properties and performance of the resulting compounds.

To conclude, this review, with its overview of the molecular tools used for the study of G4s, on one hand, testifies to the great interest that this research field has garnered in recent years; on the other hand, it highlights the importance of the topic, which will undoubtedly see further expansion with the aim of enabling the precise detection and modulation of G4 structures in live cells and will ultimately better elucidate their roles in cellular processes and disease mechanisms.
